# Interactive effects of seedling number per hill and plant spacing on source-sink dynamics and yield formation in rice

**DOI:** 10.3389/fpls.2025.1707882

**Published:** 2025-11-24

**Authors:** Xiaoyan Wu, Dongjie Xie, Anjie Xu, Pengli Yuan, Jiada Huang, Ligeng Jiang

**Affiliations:** 1 Key Laboratory of Crop Cultivation and Farming System, College of Agriculture, Guangxi University, Nanning, China; 2 Guangxi Key Laboratory of Agro-Environment and Agro-Products Safety, College of Agriculture, Guangxi University, Nanning, China

**Keywords:** Oryza sativa l., rice population structure, biomass accumulation andallocation, nitrogen and organic carbon accumulation, trait trade-offs

## Abstract

A high-quality rice population structure serves as the foundation for achieving high yields, with plant spacing and the number of seedlings per hill playing key roles in regulating this structural development. However, the combined effects of seedling number per hill and plant spacing on yield formation and resource allocation in rice (*Oryza sativa* L.) are not yet fully understood. This study aimed to assess the effects of coordinated changes in seedling number per hill and plant spacing on source-sink characteristics and yield in rice, while maintaining a constant seedling density. The study evaluated the response of four rice varieties to combined variations in seedling number per hill and plant spacing, all under a baseline seedling density of 60 × 10^4^ ha^-^¹, over a two-year field experiment. Linear models, mixed-effects models, ridge regression, and structural equation modeling were used to examine the effects of these factors on rice growth, biomass allocation, nitrogen and carbon accumulation, and yield. Results showed that increasing seedlings per hill and plant spacing reduced yield and above-ground biomass (AGB) but increased the harvest index (HI). Single seedlings with narrow spacing produced the highest yield (7.27 t ha^-^¹), AGB (16.4 t ha^-^¹), and the lowest HI (0.46). The combined effects of seedling number per hill and plant spacing negatively affected tiller number, number of effective panicles, leaf area index, and nutrient accumulation, while positively influencing specific leaf area and panicle biomass allocation. Organic carbon accumulation (43.52%) and biomass accumulation (31.32%) were major contributors to yield variation. Path analysis indicated that 96% of yield variance was accounted for, highlighting the importance of panicle biomass, organic carbon accumulation, and the source-sink balance between stems and panicles. In conclusion, single seedlings with narrow spacing (12.93 cm spacing with 1 seedling per hill) optimize the source-sink balance in rice, compensating for a lower harvest index by enhancing biomass accumulation, thereby increasing yield.

## 1 Introduction

By 2050, the global population is projected to reach 9.7 billion (Tian et al., 2016; United Nations Department of Economic and Social Affairs, 2022), driving an approximately 70% increase in food demand compared to current levels (Cole et al., 2018). Factors such as dwindling natural resources, rapid urbanization, demographic shifts, and changing diets (Beltran-Peña et al., 2020) are expected to significantly impact global food security. Rice (*Oryza sativa* L.), a staple for nearly half the world’s population (Li et al., 2018) and grown in over 100 countries (Birla et al., 2017), is critical for addressing these challenges. Modern agricultural technologies have increased rice yields from 1.86 t ha^-^¹ in 1961 to 4.66 t ha^-^¹ in 2019 (Bin Rahman and Zhang, 2023). However, despite a potential yield of 8.5 t ha^-^¹, actual yields average only 4.0 t ha^-^¹, highlighting a significant yield gap (Yuan et al., 2021). Optimizing crop management models is essential to improve rice production on existing arable land (Huang et al., 2022).

Rice production is concentrated in Asia, with China ranking as the second-largest rice-growing country by area and the largest in total production. Over the past two decades, China's rice yield has remained stable (Hollosy et al., 2023). In the middle and lower reaches of the Yangtze River, late-season rice has achieved up to 90% of its potential yield, with yield increases gradually stabilizing (Zhang et al., 2019; Rong et al., 2021). From 2018 to 2022, the average rice yield in China was 7.06 t ha^-^¹ (FAO, 2023). This reduction in the yield gap is attributed to advanced integrated crop management strategies (He et al., 2020; Pan et al., 2022; Liu et al., 2023). Enhancing rice yields relies on effectively coordinating individual plant development, population structure, and yield formation. Current research predominantly focuses on micro-level processes, such as physiological metabolism and gene expression regulation. However, population-level processes critical to yield and quality, including canopy light interception, light energy conversion, and assimilate distribution, receive less attention (Xiao et al., 2021). Research on developing high-yield rice populations and improving population-level productivity remains relatively underexplored.

Achieving high yield depends on an optimized population structure and efficient functioning. Previous studies have shown that plant spacing configurations significantly influence population structure and the development and growth of rice tillers. Dunn et al. (2020) demonstrated that within a wide range of planting densities, grain yield remained stable due to compensatory effects, such as increased tillers per plant and more grains per panicle at lower densities. At a constant planting density, wider row spacing (36 cm) resulted in lower yields compared to narrower spacing, attributed to reduced compensatory interactions among neighboring plants (Dunn et al., 2020). In contrast, the system of rice intensification, which employs wider plant spacing, promotes root growth, increases tiller numbers, boosts effective panicle numbers, and raises total leaf counts, leading to yield increases of up to 40% (Thakur et al., 2010). Optimizing plant spacing also reduces weed pressure and improves mechanized production efficiency. For instance, reducing wheat row spacing to 11 cm significantly lowered weed populations and achieved the highest grain yield of 4.91 t ha^-^¹ (Fahad et al., 2015). Advances in mechanical pot-seedling transplanting techniques now allow adjustable row spacing, improving yield potential in japonica rice. The highest yield was recorded at a transplanting density of 26.88×10^4^ plants ha^-^¹ (Hu et al., 2020). Previous research has primarily focused on optimizing plant spacing and improving resource-use efficiency for individual and population-level yield responses. However, limited studies have examined the combined effects of plant spacing configurations and the number of seedlings per hill on yield at both levels. Furthermore, research on how the interaction between seedlings per hill and plant spacing, while maintaining consistent planting density, affects photosynthate accumulation, biomass distribution, and the carbon-nitrogen balance in rice remains scarce.

Plants frequently exhibit phenotypic plasticity in response to changing environmental conditions (Wang et al., 2020; Rehling et al., 2021; Turcotte and Levine, 2016). A key aspect of this adaptation involves alterations in biomass allocation among plant organs (Kleyer et al., 2019). Biomass allocation, defined as the distribution of biomass across organs, is measured by quantifying organ biomass, reflecting allocation dynamics over time (Poorter et al., 2012). Environmental conditions significantly influence biomass coordination and distribution, with functional traits providing critical insights into these patterns (Yin et al., 2019). Traits such as organ mass, shape, surface area, and chemical composition elucidate how biomass distribution shifts in response to environmental factors (Kleyer and Minden, 2015; Kramer-Walter and Laughlin, 2017; Cebrián-Piqueras et al., 2021). These functional traits are central to understanding plant-environment interactions (Westoby and Wright, 2006; Bornette and Puijalon, 2011; Zhang et al., 2017). Trade-offs and coordination among traits are often represented by positive or negative correlations, reflecting allometric growth patterns driven by biomechanical and physiological constraints under varying conditions (Freschet et al., 2015; Tyagi and Kumar, 2024). For example, in barley, increasing planting density reduces root mass fraction but increases stem mass fraction and biomass per unit area, improving cropping efficiency. However, beyond 230 plants m^-^², the harvest index declines, indicating that higher efficiency does not always result in increased yields (Hecht et al., 2016). Environmental factors also induce trade-offs, such as drought conditions increasing root mass while reducing stem, leaf, and reproductive biomass. Annual plants respond more strongly to drought than perennial species (Eziz et al., 2017). Similarly, shade-tolerant angiosperms exhibit higher stem-specific density and seed mass but lower specific leaf area and nitrogen content than shade-intolerant species (Pavanetto et al., 2024). These variations in plant traits reliably indicate responses to environmental factors and reflect adaptations to specific constraints (Fynn et al., 2005). Trade-offs in functional traits are particularly evident in biomass accumulation and allocation, which are shaped by allocation constraints. These constraints arise from limited resources, where increasing allocation to one component necessitates reducing another (Garland et al., 2022). Trade-offs are foundational for understanding the evolution, plasticity, and constraints of phenotypes. Previous research has examined biomass allocation and environmental responses across various plant species. However, this study focuses on how the interaction between seedlings per hill and plant spacing influences functional trait trade-offs within rice populations. By investigating how adjusting stand arrangement affects key factors such as rice biomass accumulation, nitrogen and organic carbon stocks, carbon-nitrogen balance, and grain yield, this study provides valuable insights for optimizing agronomic practices to enhance productivity. The findings offer guidance for more sustainable and efficient rice cultivation practices, contributing to efforts to meet the growing global food demand. Specifically, it examines how variations in the number of seedlings per hill and plant spacing affect rice growth, biomass accumulation and allocation, nitrogen and organic carbon accumulation, carbon-nitrogen balance, and grain yield. The experiments were conducted at a seedling density of 60×10^4^ plants per hectare. By adjusting the combinations of seedling number per hill and plant spacing, four rice varieties (two hybrid and two conventional types) were selected to ensure the robustness and reliability of the experimental outcomes.

The study has three objectives: (1) to evaluate how coordinated adjustments in the number of seedlings per hill and plant spacing impact rice growth, material accumulation and allocation, carbon-nitrogen balance, and yield; (2) to explore the trade-offs between functional traits and their effects on yield formation under varying conditions; and (3) to analyze the mechanisms of yield formation and strategies for optimizing biomass allocation in rice.

## 2 Materials and methods

### 2.1 Experimental site

**Figure 1:**
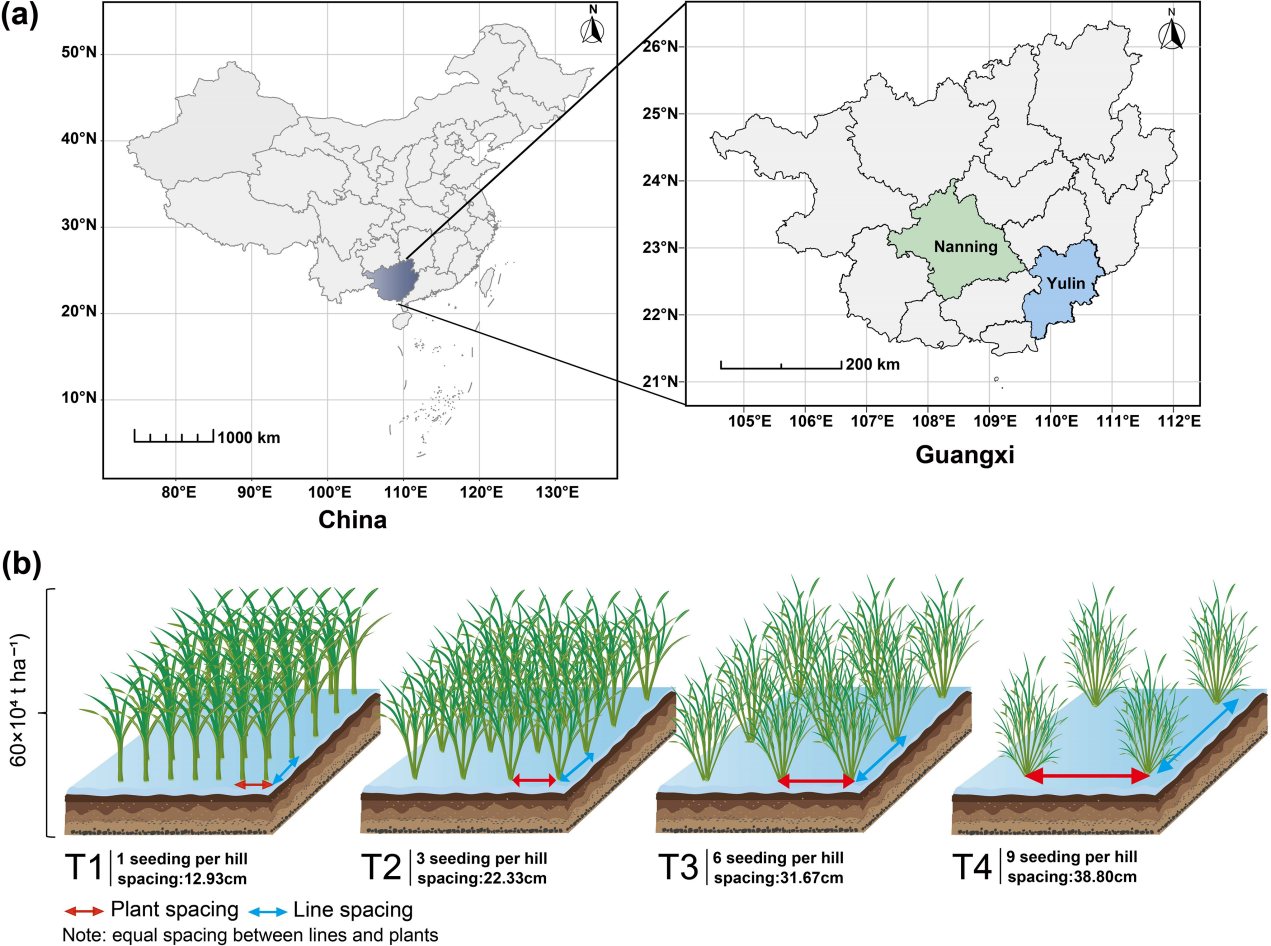
Map of Guangxi, China **(a)** and rice planting configurations **(b)**. Panel **(a)**: Experimental sites were the Guangxi University experimental station in Nanning (22°85′N, 108°28′E) and the Dali Town experimental site in Yulin (22°74′N, 110°18′E). Panel **(b)**: Four rice planting configurations: T1, 12.93 cm spacing with 1 seedling per hill. T2, 22.33 cm spacing with 3 seedlings per hill. T3, 31.67 cm spacing with 6 seedlings per hill. T4, 38.80 cm spacing with 9 seedlings per hill.

The experiment was conducted from March to November 2020 at the Guangxi University Experiment Station in Nanning, China (22°85′N, 108°28′E), covering both the early and late seasons. In the late season of 2021, the experiment was simultaneously conducted at both the Guangxi University Experiment Station in Nanning and the Dali Town Experiment Station in Yulin (22°74′N, 110°18′E) (**Figure 1a**). During the 2020 growing seasons, the average temperatures were 24.42°C in the early season and 27.0°C in the late season, with total precipitation of 579.0 mm and 664.1 mm, respectively. In the late season of 2021, the average temperatures in Nanning and Yulin were 27.4°C and 26.5°C, respectively, with corresponding precipitation totals of 587.8 mm and 671.3 mm. The physicochemical properties of the soil in the 0~20 cm layer are provided in the supplementary material (**Supplementary Table S1**). Daily temperature and precipitation data for Nanning in 2020, as well as for both Nanning and Yulin in 2021, were obtained from the Xihe Energy Network (https://www.xihe-energy.com) (**Supplementary Figure S1**).

### 2.2 Experimental design

In 2020, transplanting was used, while direct seeding was employed in 2021. The basic density, fertilizer application rate and experimental design of direct-seeded rice and transplanted rice are consistent. The experiment followed a two-factor split-plot design, with rice variety as the main plot factor and coordinated variations in seedling number per hill and plant spacing under equal density as the subplot factor. Four rice varieties were used: two conventional varieties (Zhenguiai and Guiyu 9) and two hybrid varieties (Zhuangxiangyoubaijin5 and Yexiangyou 2). The base density for each variety was 60 × 10^4^ ha^-^¹. The experimental treatments varied the number of seedlings per hill and plant spacing, with the following combinations: T1 used one seedling per hill with 12.93 cm × 12.93 cm spacing, T2 used three seedlings per hill with 22.33 cm × 22.33 cm spacing, T3 used six seedlings per hill with 31.67 cm × 31.67 cm spacing, and T4 used nine seedlings per hill with 38.80 cm × 38.80 cm spacing (**Figure 1b**). In this study, 'treatment' refers to the combined effect of seedling number per hill and plant spacing. Each treatment was replicated three times, yielding a total of 48 plots. Detailed information, including the plot area and the total number of holes in each plot, is provided in **Supplementary Table S2**. Fertilizer application was as follows: 180 kg ha^-^¹ of nitrogen (N), 180 kg ha^-^¹ of potassium oxide (K^2^O), and 90 kg ha^-^¹ of phosphorus pentoxide (P_2_O_5_). Nitrogen and potassium fertilizers (urea and potassium chloride, respectively) were applied in a 5:3:2 ratio at three stages: basal, tillering, and heading. Phosphorus (superphosphate) was applied entirely at the basal stage. Fertilizers were applied two days before transplanting, 10 days after transplanting (tillering stage), and approximately 30 days after transplanting (heading stage). Pesticides were applied as necessary, with careful attention to minimizing their impact on the experimental outcomes.

### 2.3 Sampling and measurement

Mature above-ground rice plants were harvested to assess biomass and trait data (**Supplementary Table S6**). Three representative hills from each plot were selected, and the number of tillers and effective panicles per hill were recorded. The plants were then separated into three parts: stems (including stalks and leaf sheaths), leaves, and panicles. Leaf area was estimated using the length-width coefficient method based on 20 rice leaves to calculate the specific leaf area (SLA) and leaf area index (LAI). All plant parts were first dried at 105°C for 30 minutes, followed by drying at 85°C to a constant weight. Dry matter content was determined by weighing the samples. Above-ground biomass (AGB) was calculated as the sum of the stem, leaf, and panicle biomass per unit area. The biomass of the stem, leaf, and panicle (kg ha^-^¹) was calculated using the following formula: Stem/leaf/panicle biomass (kg ha^-^¹) = dry matter weight per hill (stem/leaf/panicle) × total number of hills per plot / total plot area. Biomass allocation was assessed by calculating the stem mass fraction (SMF), leaf mass fraction (LMF), and panicle mass fraction (PMF), which represent the proportion of stems, leaves, and panicles in the total above-ground biomass, respectively.

Yield measurement: All rice plants within each plot, excluding the sampled plants, were harvested. Fresh weight was measured after weeds and empty grains were removed through air separation. The moisture content of the rice grains was determined using a grain moisture meter (KETTPM8188A, Japan), and the grain dry weight was calculated based on a standard moisture content of 13.5%. Yield per unit area was calculated as the dry weight of harvested rice grains per plot divided by the adjusted plot area (total plot area minus the area of the three sampled rice plots). The harvest index (HI) was calculated as the ratio of economic yield to above-ground biomass. Dried samples of stems, leaves, and panicles were analyzed for organic carbon and total nitrogen content. Organic carbon was assessed using the potassium dichromate volumetric method with external heating. The samples were crushed and digested with sulfuric acid (H^2^SO^4^) and hydrogen peroxide (H^2^O^2^), followed by analysis with an AA3 continuous flow analyzer (SEAL AutoAnalyzer 3, Germany). Nitrogen accumulation in the stems, leaves, and panicles was calculated as described by Wu et al. (2022).

N accumulation by stem (SNA, kg ha^-1^) = N content of stem (g kg^-1^) × stem weight (kg ha^-1^) / 1000.

N accumulation by leaf (LNA, kg ha^-1^) = N content of leaf (g kg^-1^) × leaf weight (kg ha^-1^) / 1000.

N accumulation by panicle (PNA, kg ha^-1^) = N content of panicle (g kg^-1^) × panicle weight (kg ha^-1^) / 1000.

Similarly, organic carbon accumulation was determined using the formula: organic carbon content (g kg^-1^)× ODM (kg ha^-1^) / 1000, where ODM is the total dry matter weight of the panicles, leaves, and stems.

### 2.4 Statistical analysis

This study investigated the effects of rice variety (Variety) and coordinated changes in seedling number per hill and plant spacing under equal density (Treatment) on above-ground traits and yield. The combined changes in seedling number and plant spacing were considered as the 'Treatment' factor. Due to the experimental design, the difference between the 2020 transplanting trial and the 2021 direct seeding trial is equivalent to the inter-annual difference. Therefore, separate variance analyses were performed for transplanting and direct seeding rice. A two-way ANOVA was performed to assess the effects of variety and treatment on rice growth. Normality was tested using the Shapiro-Wilk test, and for traits that were not normally distributed (*p* < 0.05), PERMANOVA was applied using the adonis () function in R. Pairwise *post-hoc* comparisons were conducted using the emmeans test with Bonferroni correction. After the separate analyses, a linear mixed-effects model was employed to justify merging the datasets. Planting method, site, and season were treated as random effects (Random = ~ (1 | Planting) + (1 | Site) + (1 | Season)), while cultivar and treatment were considered fixed effects (Fixed = ~ 1 + Variety * Treatment). This integrated approach allowed for the evaluation of trends across planting methods and addressed potential cross-year effects.

A linear model was used to examine the impact of above-ground growth traits and biomass accumulation on yield, with the coordination between seedling number per hill and plant spacing as explanatory variables. The analysis proceeded in two stages: (1) a linear model was constructed for each trait, including all interaction effects related to yield, retaining only statistically significant traits or interactions; and (2) a comprehensive model was developed using the selected traits and interaction effects, identifying the best predictive model for yield through the dredge() function from the 'MuMIn' package (**Appendix Table S5**, where the first model is the best and only models with an Akaike Information Criterion (AIC) threshold of ΔAICc ≤ 2 are shown). To address high multicollinearity among trait variables, ridge regression was applied, incorporating regularization terms to reduce multicollinearity and enhance the accuracy of model parameters. Finally, the 'rdacca.hp' package was used to decompose the linear model and assess the contribution of various traits to yield and population productivity.

Piecewise structural equation modeling (pSEM) was employed to evaluate the proposed model concerning the effects of seedling number per hill and plant spacing on above-ground traits and rice yield, using the 'piecewiseSEM' package. To mitigate high collinearity, principal component analysis was applied to analyze biomass and organic carbon accumulation in stems, leaves, and panicles. The first two principal components were selected for inclusion in the structural equation model: PC1, which represents the overall variation in biomass and organic carbon across these plant parts, and PC2, which emphasizes the differences in grain biomass and organic carbon accumulation relative to other parts (**Supplementary Figure S5**). **Supplementary Figure S6** illustrates the composite variables for the various trait types used in the model. To optimize the model structure, treatment was treated as a grouping variable, and composite variables were constructed based on the categories of above-ground traits, which served as predictor variables. Collinearity among the composite variables was assessed using the vif () function from the 'car' package, where a variance inflation factor (VIF) value of less than 10 indicated an acceptable level of collinearity. All analyses were performed using R version 4.3.2 (R Development Core Team, 2024).

## 3 Results

### 3.1 Effects of variety and treatment on rice yield, above-ground biomass, and harvest index

**Figure 2:**
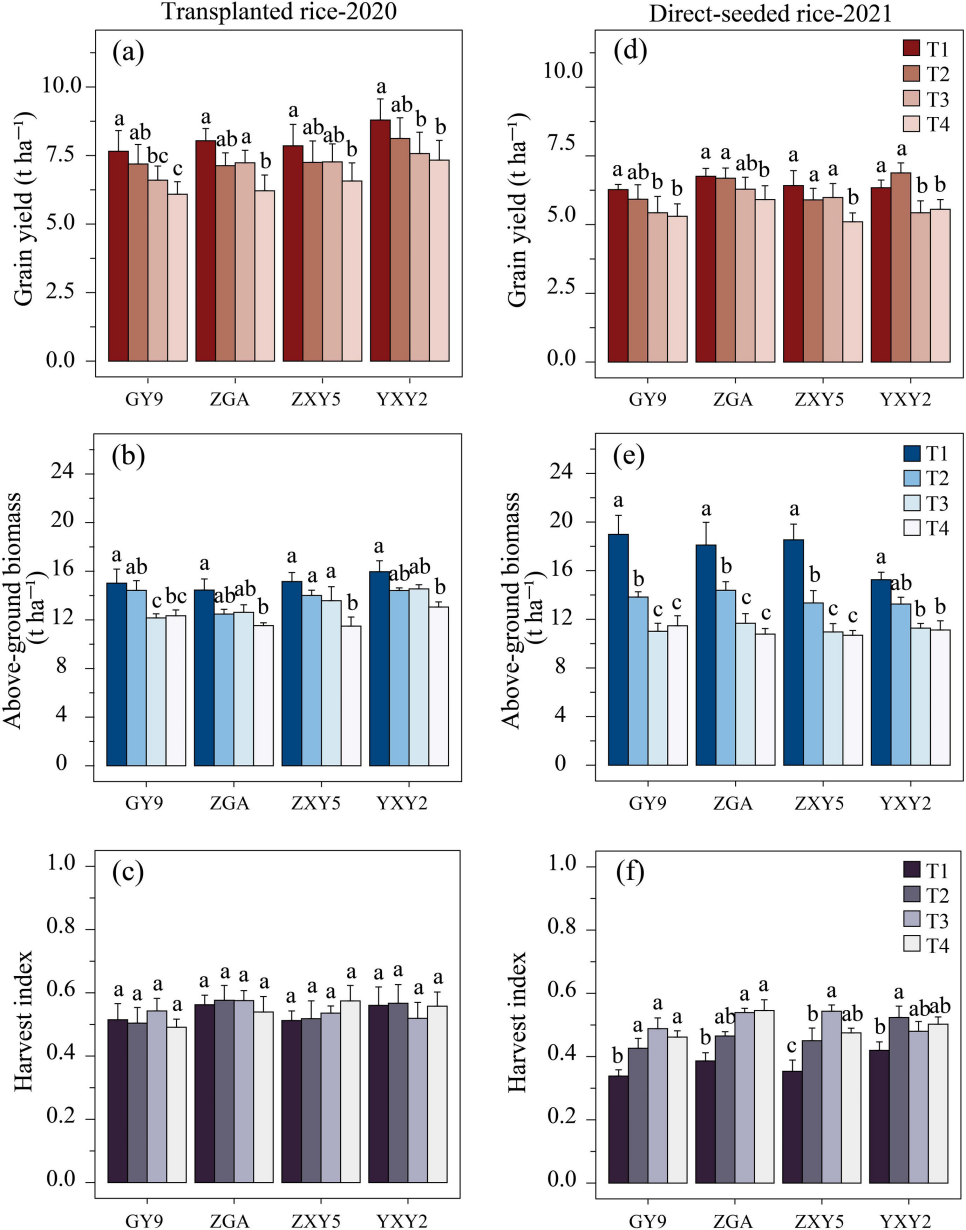
Effects of varieties and treatments on yield, above-ground biomass, and harvest index of transplanted rice **(a, b, c)** and direct-seeded rice **(d, e, f)**. GY9, Guiyu 9. ZGA, Zhenguiai. ZXY5, Zhuangxiangyoubaijin 5. YXY2, Yexiangyou 2. T1, 12.93 cm spacing with 1 seedling per hill. T2, 22.33 cm spacing with 3 seedlings per hill. T3, 31.67 cm spacing with 6 seedlings per hill. T4, 38.80 cm spacing with 9 seedlings per hill. Different lowercase letters represent significant differences among treatments (*p* < 0.05).

We used ANOVA to assess the effects of rice variety and treatment (the combined effects of seedling number per hill and plant spacing) on rice yield, above-ground biomass (AGB), and harvest index (HI). Additionally, the impact of random effects, such as planting method, growing season, and experimental site, was evaluated using linear mixed-effect models. The ANOVA results showed that both rice variety and treatment significantly influenced yield and AGB (**Supplementary Table S4**). In transplanted rice, the hybrid rice varieties ZXY5 and YXY2 achieved the highest yields, at 7.23 t ha^-^¹ and 7.95 t ha^-^¹, respectively. In direct-seeded rice, the conventional rice variety ZGA produced the highest yield, at 6.41 t ha^-^¹. Linear mixed models further revealed that the treatment significantly reduced both yield and AGB (β_yield = –1.265, β_AGB = –5.617, *p* < 0.001, **Supplementary Table S3**), while significantly increasing HI (β_HI = 0.076, *p* < 0.01), suggesting an inverse relationship between these variables. *Post hoc* tests (**Supplementary Table S4**) indicated that increased plant spacing and more seedlings per hill led to a significant decrease in yield (**Figure 2a, d**), with a similar trend observed for AGB (**Figure 2b, e**). Conversely, HI exhibited an opposite trend in direct-seeded rice. However, no significant difference in HI was observed among the treatments for transplanted rice (**Figure 2e, f**). For transplanted rice, T1 produced the highest yield (8.09 t ha^-^¹) and AGB (15.15 t ha^-^¹), outperforming treatments T2, T3, and T4. Notably, T1 increased yield by 23.43% compared to T4, although no significant differences were observed in HI across treatments (range: 0.49~0.57). For direct-seeded rice, T1 achieved a yield of 6.45 t ha^-^¹ and AGB of 17.72 t ha^-^¹, representing increases of 17.92% and 60.87%, respectively, over T4. Furthermore, T4 showed a 32.52% increase in HI compared to T1. Overall, this two-year study demonstrates that the T1 treatment provides the highest yield potential, underscoring its practical significance for rice cultivation.

### 3.2 Effects of variety and treatment on growth traits

**Figure 3:**
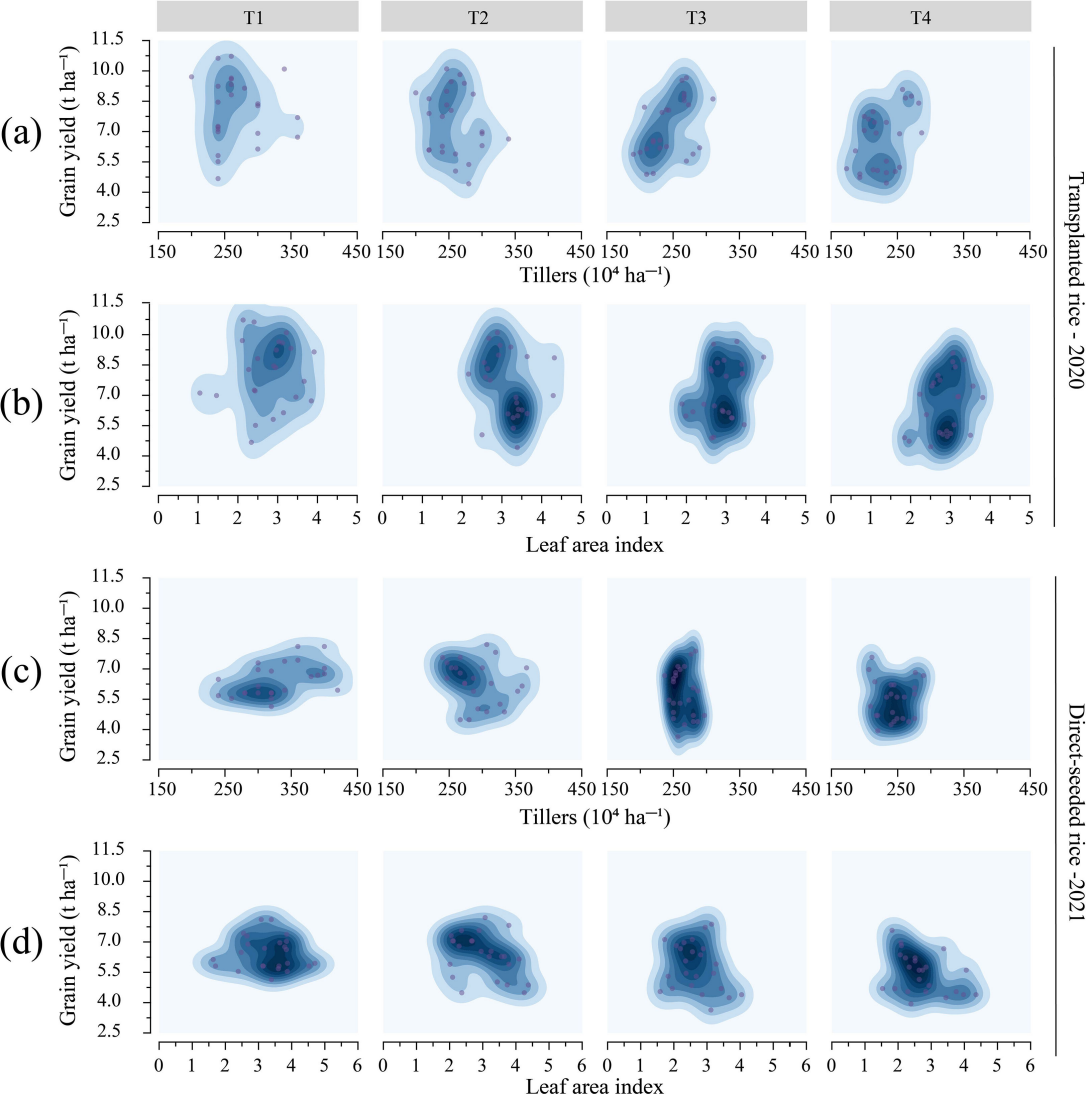
Effects of tiller number and leaf area index on rice yield under different treatments. **(a, b)** demonstrate the effects of different treatments on tiller number and LAI in transplanted rice; **(c, d)** emphasize the effects of different treatments on tiller number and LAI in direct-seeded rice. T1, 12.93 cm spacing with 1 seedling per hill. T2, 22.33 cm spacing with 3 seedlings per hill. T3, 31.67 cm spacing with 6 seedlings per hill. T4, 38.80 cm spacing with 9 seedlings per hill. Dark areas indicate dense data points, and light areas indicate sparse data points.

Variety and treatment (the combined effects of seedling number per hill and plant spacing) significantly influenced the number of tillers, effective panicle number (NEP), and leaf area index (LAI) in rice (**Supplementary Table S4**, **Supplementary Figure S2**, **Supplementary Figure S3**, *p* < 0.05). Treatment negatively affected tiller number, NEP, and LAI, while positively influencing specific leaf area (SLA) (**Supplementary Table S4**). As plant spacing and seedling number per hill increased, both tiller number and NEP decreased. In transplanted rice, T1 resulted in a tiller number of 268.33 × 10^4^ ha^-^¹ and NEP of 245.0 × 10^4^ ha^-^¹, representing increases of 17.52% and 12.74%, respectively, compared to T4. In direct-seeded rice, T1 showed a tiller number of 328.75 × 10^4^ ha^-^¹, NEP of 307.5 × 10^4^ ha^-^¹, and LAI of 3.27, which were 34.67%, 34.75%, and 20.18% higher than those of T4, respectively (**Supplementary Figure S2**, **Supplementary Figure S3**). According to the 2D density map (**Figure 3**), for T1 and T2 treatments, transplanted rice had tiller numbers ranging from 200~350 × 10^4^ ha^-^¹, with yields between 7.5~10.5 t ha^-^¹, whereas direct-seeded rice had tiller numbers of 250~400 × 10^4^ ha^-^¹ and yields of 5.5~7.5 t ha^-^¹. The data points for T3 and T4 were more clustered, with yield decreasing as tiller number declined. Differences in LAI among treatments were minimal. Among all varieties, YXY2 exhibited the highest values for tiller number, NEP, LAI, and SLA. These findings suggest that T1 (single seedling per hill, plant spacing of 12.93 cm) effectively enhanced rice growth by promoting increases in tiller number, NEP, and LAI.

### 3.3 Effects of variety and treatment on biomass accumulation and allocation across different organs

**Figure 4:**
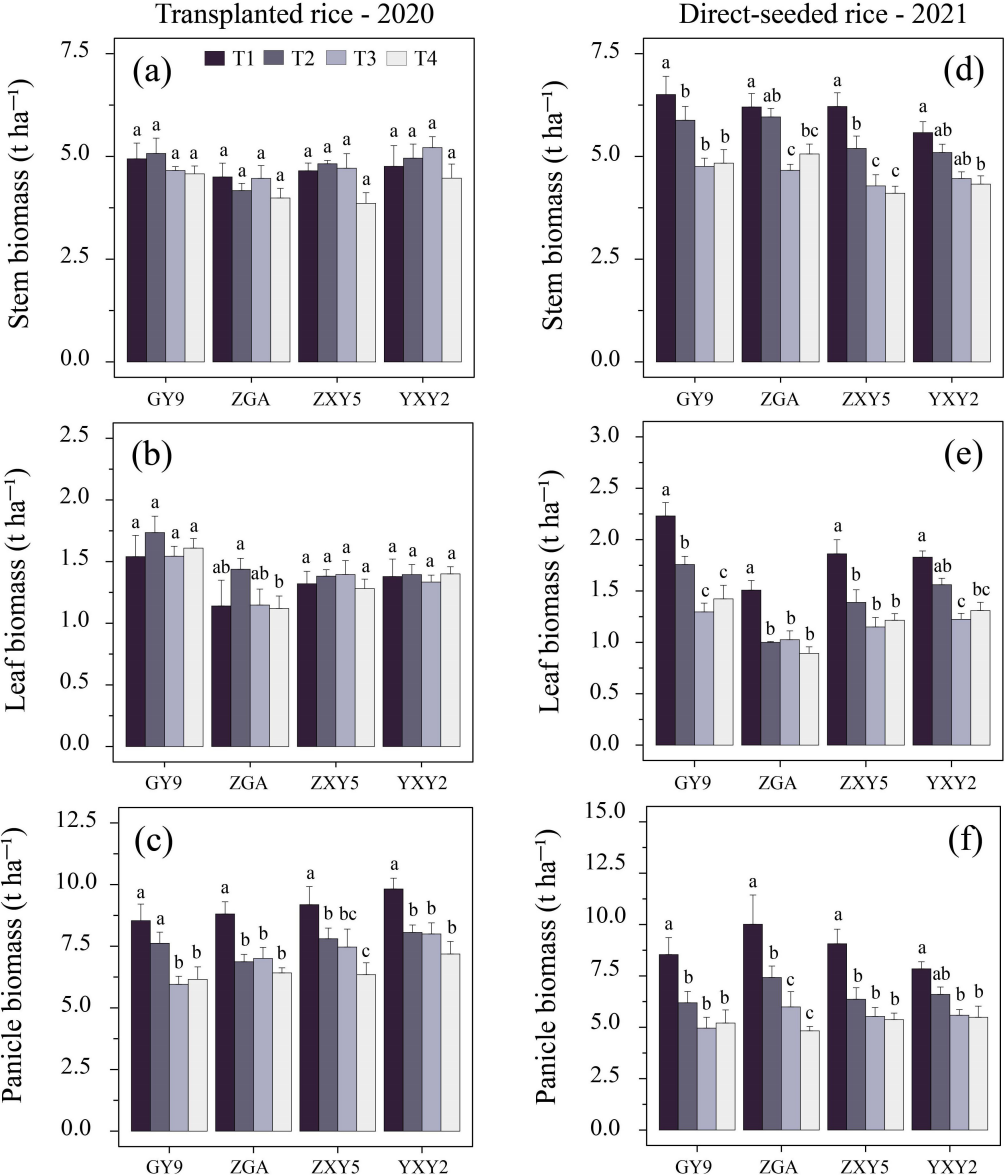
Effects of variety and treatment on stem, leaf, panicle biomass in rice. **(a–c)** demonstrate the effects of different treatments on stem, leaf, panicle biomass in transplanted rice, **(d–f)** emphasize the effects of different treatments on stem, leaf, panicle biomass in direct-seeded rice. GY9, Guiyu 9. ZGA, Zhenguiai. ZXY5, Zhuangxiangyoubaijin 5. YXY2, Yexiangyou 2. T1, 12.93 cm spacing with 1 seedling per hill. T2, 22.33 cm spacing with 3 seedlings per hill. T3, 31.67 cm spacing with 6 seedlings per hill. T4, 38.80 cm spacing with 9 seedlings per hill. Different lowercase letters represent significant differences among treatments (*p* < 0.05).

**Figure 5:**
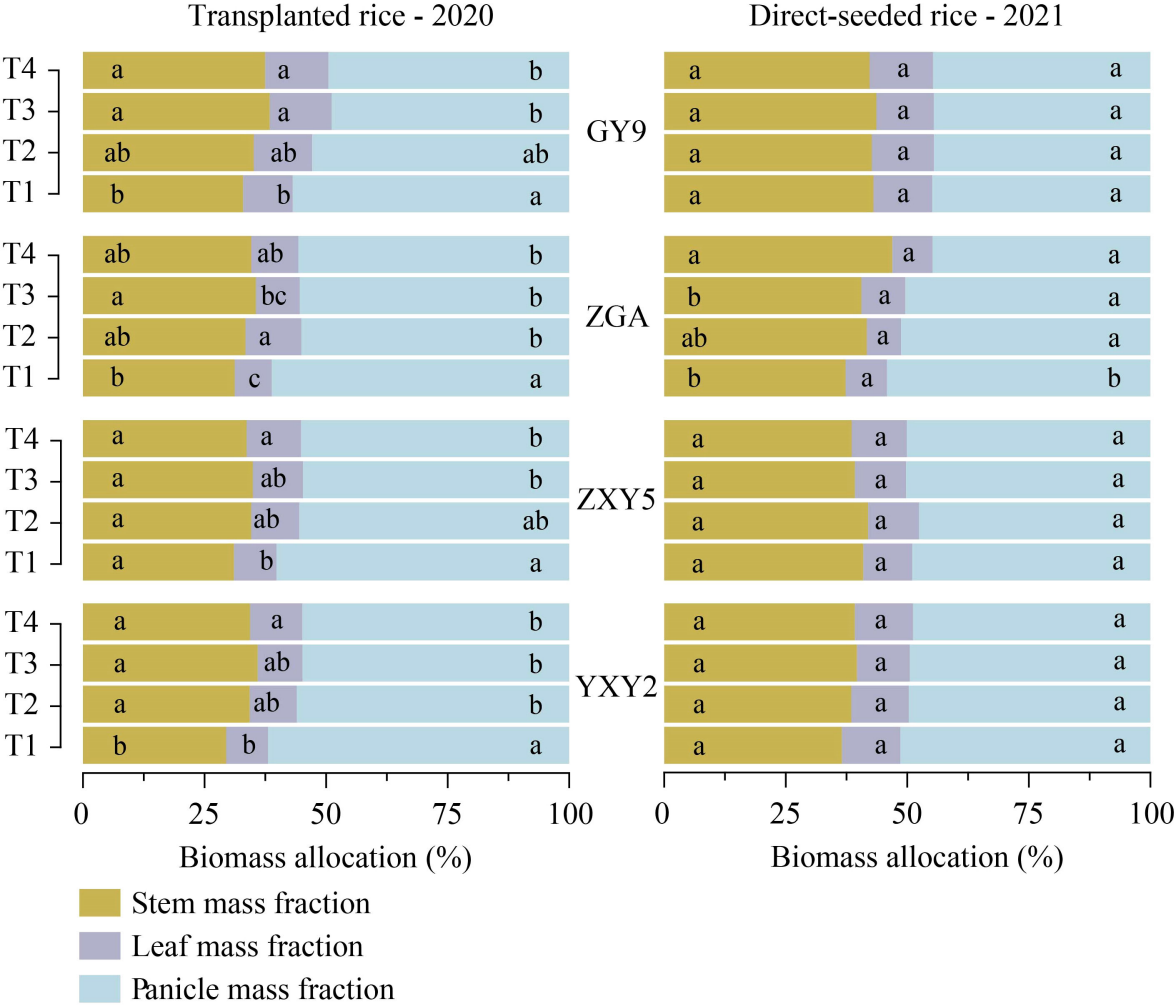
Effects of variety and treatment on stem, leaf, panicle biomass allocation in rice. GY9, Guiyu 9. ZGA, Zhenguiai. ZXY5, Zhuangxiangyoubaijin 5. YXY2, Yexiangyou 2. T1, 12.93 cm spacing with 1 seedling per hill. T2, 22.33 cm spacing with 3 seedlings per hill. T3, 31.67 cm spacing with 6 seedlings per hill. T4, 38.80 cm spacing with 9 seedlings per hill. Different lowercase letters represent significant differences among treatments (*p* < 0.05).

The combined effect of seedling number per hill and plant spacing significantly influenced biomass accumulation and allocation patterns in rice (**Supplementary Table S4**). A simultaneous increase in both factors reduced stem biomass (SB), leaf biomass (LB), panicle biomass (PB), and panicle mass fraction (PMF), while increasing stem mass fraction (SMF) and leaf mass fraction (LMF) (**Figure 4**, **Supplementary Table S3**, *p* < 0.05). In transplanted rice, T1 resulted in significant increases in SB and PB by 11.59% and 39.26%, respectively, compared to T4 (SB = 4.71 t ha^-^¹, PB = 9.09 t ha^-^¹). In direct-seeded rice, T1 exhibited increases in SB, LB, and PB by 52.71%, 53.47%, and 69.74%, respectively, compared to T4 (SB = 7.0 t ha^-^¹, LB = 1.86 t ha^-^¹, PB = 8.87 t ha^-^¹) (**Supplementary Table S4**). As the number of seedlings per hill and plant spacing increased, SMF and LMF tended to rise, while PMF decreased. This difference was statistically significant in transplanted rice (**Figure 5**). In transplanted rice, the YXY2 variety exhibited the highest panicle biomass accumulation, whereas in direct-seeded rice, GY9 accumulated the greatest above-ground biomass. The T1 treatment, characterized by higher SB, LB, PB, and PMF, enhanced above-ground biomass, providing a strong foundation for improving yield productivity.

### 3.4 Effects of variety and treatment on nitrogen, organic carbon accumulation and carbon-nitrogen ratio

**Figure 6:**
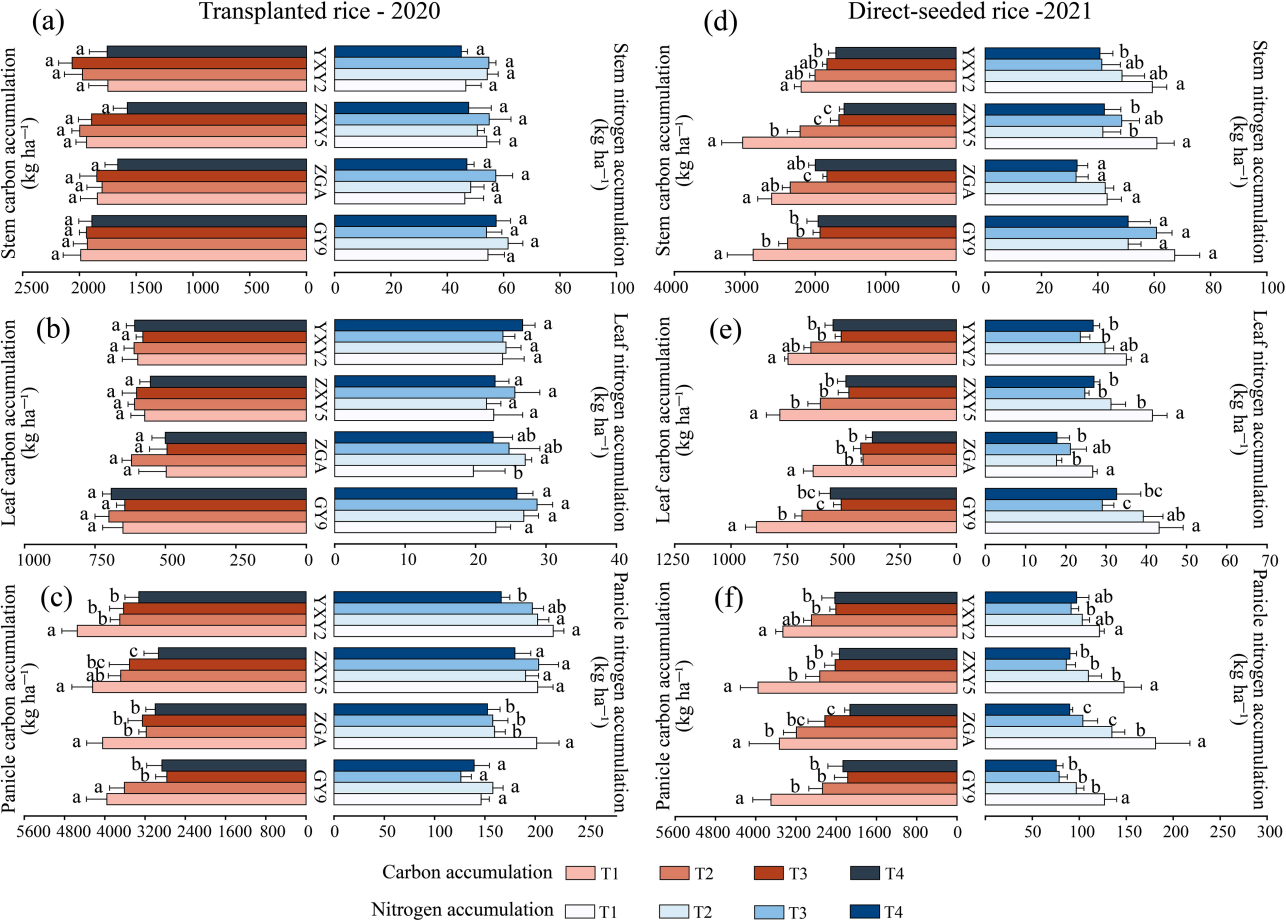
Effects of variety and treatment on nitrogen accumulation and organic carbon accumulation in stem, leaf, panicle. GY9, Guiyu 9. ZGA, Zhenguiai. ZXY5, Zhuangxiangyoubaijin 5. YXY2, Yexiangyou 2. T1, 12.93 cm spacing with 1 seedling per hill. T2, 22.33 cm spacing with 3 seedlings per hill. T3, 31.67 cm spacing with 6 seedlings per hill. T4, 38.80 cm spacing with 9 seedlings per hill. Different lowercase letters represent significant differences among treatments (*p* < 0.05).

The combined effect of seedling number per hill and plant spacing significantly reduced stem nitrogen accumulation (SNA), leaf nitrogen accumulation (LNA), and panicle nitrogen accumulation (PNA) (β_SNA = –4.85, β_LNA = –4.28, β_PNA = –47.28, *p* < 0.05; **Supplementary Table S3**). In transplanted rice, the panicle nitrogen accumulation (PNA) of ZGA and YXY2 significantly decreased with the increase in seedlings per hill and plant spacing (**Figure 6**). Specifically, compared to T1, the PNA under the T4 treatment decreased by 24.31% (ZGA) and 23.76% (YXY2), respectively. In direct-seeded rice, SNA, LNA, and PNA all showed significant decreases as seedling number and plant spacing increased. Significant differences in nitrogen accumulation were also observed among varieties. These findings indicate that the T1 treatment resulted in relatively higher nitrogen accumulation in stems, leaves, and panicles. Furthermore, the number of seedlings per hill and plant spacing also significantly influenced organic carbon accumulation in above-ground organs (**Supplementary Table S4**). As both factors increased, stem carbon accumulation (SCA), leaf carbon accumulation (LCA), and panicle carbon accumulation (PCA) generally declined. Notably, in direct-seeded rice, T4 showed reductions in SCA, LCA, and PCA by 55.17%, 54.98%, and 69.38%, respectively, compared to T1 (**Figure 6**), indicating a substantial decrease in carbon accumulation under T4. In the transplanted rice, the stem carbon-to-nitrogen ratio (SCN) of GY9, ZXY5, and YXY2 showed no significant differences among the different treatments. However, the panicle carbon-to-nitrogen ratio (PCN) decreased with the increase in plant spacing and seedlings per hill. The difference between T1 and T4 was significant in GY9 and ZXY5 varieties (**Supplementary Figure S4**). SCN in direct-seeded rice were generally higher than those in transplanted rice, suggesting greater carbon allocation to stems in direct-seeded rice. Similar to transplanted rice, T1 and T2 treatments generally showed higher LCN values in direct-seeded rice. The PCN of direct-seeded rice decreased with increasing seedling number per hill and plant spacing. Specifically, the PCN of T4 was 8.76% (ZGA), 4.79% (ZXY5), and 10.56% (YXY2) lower than that of T1. Significant differences in carbon and nitrogen accumulation were observed between conventional and hybrid rice varieties under both transplanting and direct seeding methods (**Supplementary Table S4**). In transplanted rice, GY9 exhibited the highest SNA and LNA but the lowest PNA. In contrast, hybrid rice varieties ZXY5 and YXY2 had significantly higher PNA than the two conventional rice varieties. In direct-seeded rice, ZGA exhibited the lowest SNA and LNA among the four varieties but the highest PNA.

### 3.5 Best models and pathways for influencing yield, AGB, and HI

**Figure 7:**
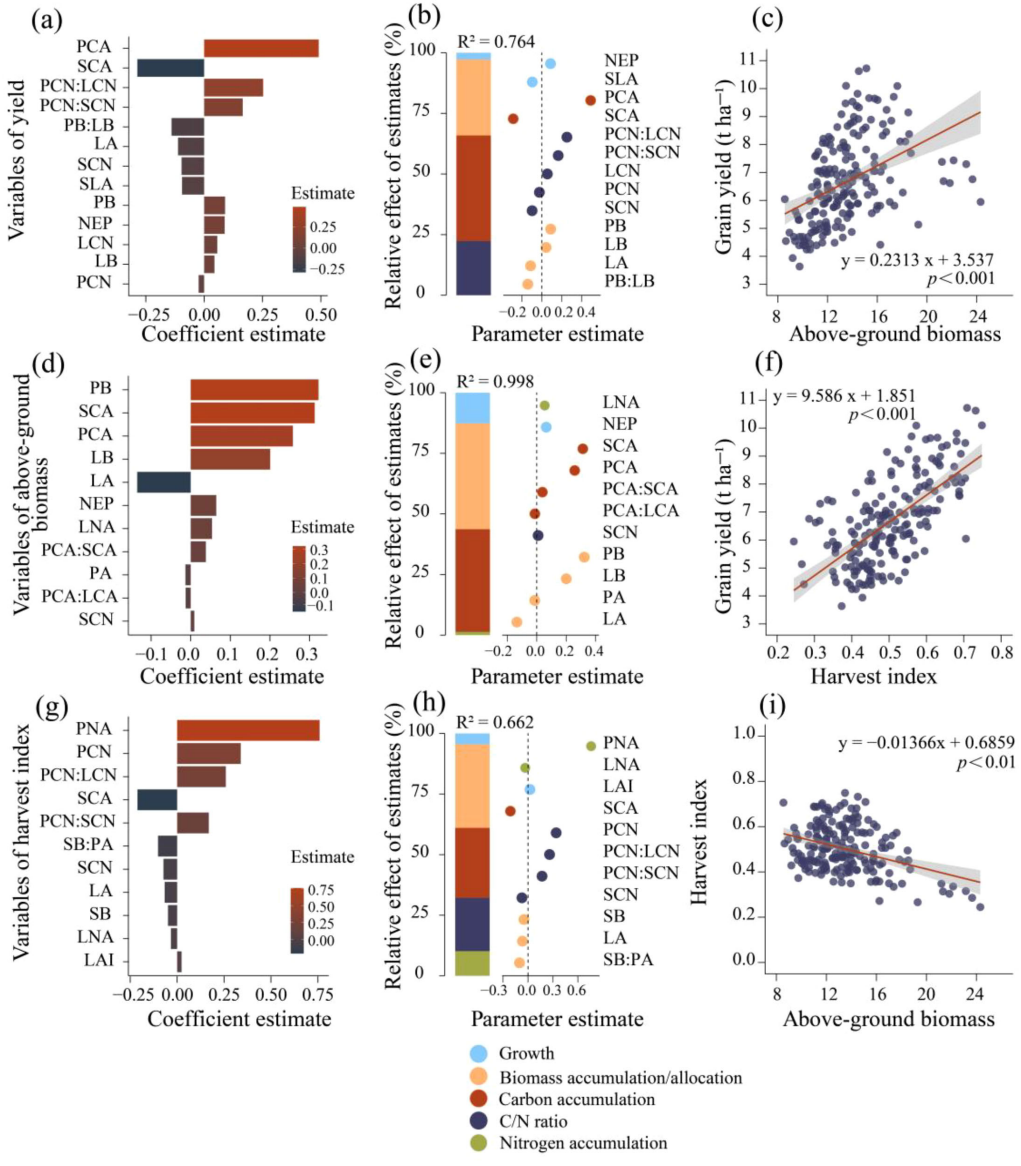
Optimal predictive models and linear regression analysis of factors influencing rice yield **(a, b)**, above-ground biomass (AGB) **(d, e)**, and harvest index (HI) **(g, h)**. **(a, d, g)** shows the importance ranking of above-ground trait variables in the ridge regression model for yield, AGB, and HI, respectively. **(b, e, h)** present the average parameter estimates (standardized regression coefficients) for the predictor variables in the ridge regression model, along with the relative importance of each predictor expressed as a percentage of explained variance. This figure illustrates the best models selected based on ΔAICc (refer to models in the SI appendix, **Supplementary Table S5**). NEP, number of effective panicles, LAI, leaf area index, SLA, specific leaf area, SB, stem biomass, LB, leaf biomass, PB, panicle biomass, SMF, stem mass fraction, LMF, leaf mass fraction, PMF, panicle mass fraction, SNA, stem nitrogen accumulation, LNA, leaf nitrogen accumulation, PNA, panicle nitrogen accumulation, SCA, stem organic carbon accumulation, LCA, leaf organic carbon accumulation, PNA, panicle organic carbon accumulation, SCN, stem carbon-nitrogen ratio, LCN, leaf carbon-nitrogen ratio, PCN, panicle carbon-nitrogen ratio.

The optimal predictive models for yield, above-ground biomass (AGB), and harvest index (HI) identify the traits most strongly associated with these outcomes (**Figure 7**, **Supplementary Table S5**). According to the models, organic carbon accumulation explains the largest proportion of yield variance (43.52%), followed by biomass accumulation and allocation (31.32%), and the carbon-nitrogen ratio (22.37%) (**Figure 7b**). Importantly, the key traits influencing yield are predominantly located in the stem and panicle (**Figure 7a**). Growth traits, along with biomass accumulation, nitrogen accumulation, and the carbon-nitrogen ratio, explain a substantial proportion of AGB variance (R² = 0.998). Specifically, biomass accumulation and allocation contribute 43.72% to AGB model variance, while organic carbon accumulation accounts for 42.23% (**Figure 7e**). In the HI model (**Figure 7h**), biomass and organic carbon accumulation are significant contributors, explaining 34.49% and 28.87% of the variance, respectively.

**Figure 8:**
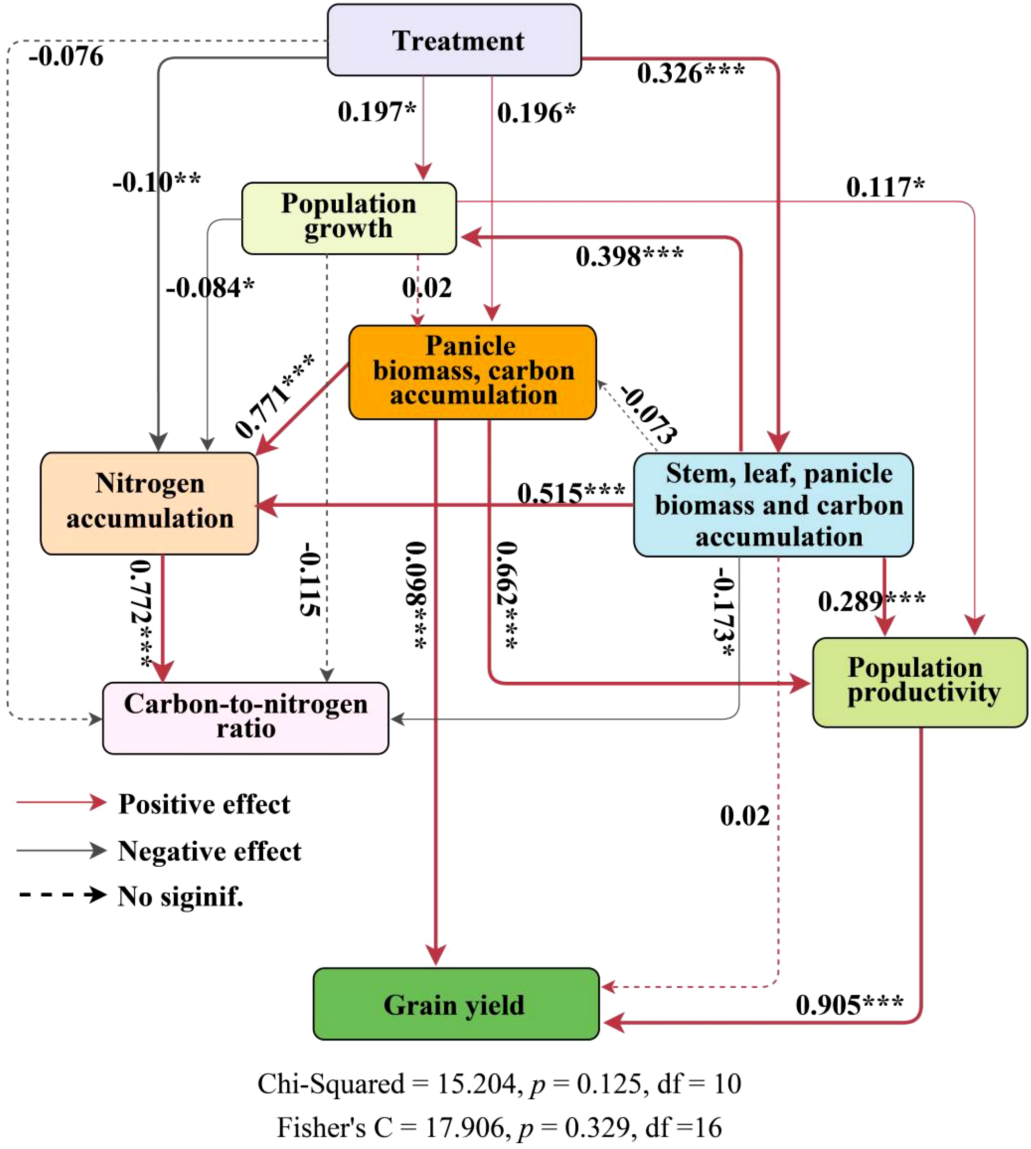
Path analysis of the effects of rice growth, biomass accumulation in various parts, nitrogen accumulation, organic carbon accumulation, carbon-nitrogen ratio, total biomass accumulation, and material transport efficiency on yield. ‘Panicle biomass, carbon accumulation’ and ‘Stem, leaf panicle biomass and carbon accumulation’ represent PC2 and PC1, respectively, from the principal component analysis of stem, leaf, grain biomass, and organic carbon accumulation (**Supplementary Figure S5**). Population growth is a composite variable consisting of tiller number, number of effective panicles, leaf area index, and specific leaf area. Nitrogen accumulation is a composite variable consisting of stem nitrogen accumulation, leaf nitrogen accumulation, and panicle nitrogen accumulation. Carbon-to-nitrogen Ratio is a composite variable consisting of stem carbon-nitrogen ratio, leaf carbon-nitrogen ratio, and panicle carbon-nitrogen ratio. Population productivity is a composite variable consisting of above-ground biomass and harvest index. Dashed arrows indicate statistically insignificant relationships between variables. Gray solid arrows and red solid arrows indicate significant negative and positive relationships, respectively. ***, *p* < 0.001; **, *p* < 0.01; *, *p* < 0.05.

The structural equation model (**Figure 8**) examines both direct and indirect relationships among key rice growth traits—biomass accumulation, organic carbon accumulation, nitrogen accumulation, the carbon-nitrogen ratio, above-ground biomass (AGB), and harvest index (HI)—in relation to yield, accounting for 96% of the variation in yield. Principal component analysis (**Supplementary Figure S5**) identified two distinct strategies for rice material allocation: one involves the simultaneous accumulation of biomass and organic carbon across stems, leaves, and panicles (PC1), while the other focuses solely on panicle accumulation (PC2). Within the structural equation model, PC2 plays a pivotal role. Both PC2 and population productivity have significant direct effects on yield, with path coefficients of 0.098 and 0.905 (*p* < 0.001), respectively. the number of seedlings per hill and plant spacing (treatments) indirectly influence yield by affecting panicle biomass and organic carbon accumulation (PC2), as well as overall biomass and carbon accumulation across stems, leaves, and panicles (PC1), with an indirect effect of 0.233. The strongest direct effect on yield is attributed to population productivity, with a coefficient of 0.905 (**Supplementary Figure S6**). Furthermore, panicle biomass and organic carbon accumulation exert indirect effects on the carbon-nitrogen ratio through their influence on nitrogen accumulation. To further investigate the relationship between material accumulation and allocation strategies in rice panicles and the carbon-nitrogen ratio in above-ground components, linear regression analyses were conducted on PC1, PC2, and the carbon-nitrogen ratios of different plant parts (**Supplementary Figure S7**). A statistically significant linear relationship was observed: for each unit increase in SCN and PCN, PC2 decreased by 0.021 and 0.097 units, respectively. In contrast, LCN showed a positive correlation with PC2 (r = 0.425, *p* < 0.001).

## 4 Discussion

### 4.1 Effects of seedling number per hill and plant spacing on source-sink characteristics and yield in rice

This experiment examined the combined effects of the number of seedlings per hill and plant spacing affected source-sink traits and yield in rice, while maintaining a constant seedling density of 60 × 10^4^ ha^-^¹. With a constant density, increasing plant spacing (and the number of seedlings per hill) significantly reduced yield and above-ground biomass (AGB) but increased the harvest index (HI) (**Figure 2**). Among the treatments, T1(12.93 cm spacing with 1 seedling per hill) produced the highest yield and AGB but exhibited a relatively low HI. Linear regression analysis showed that yield was positively correlated with both AGB and HI. Specifically, a 0.1 unit increase in HI corresponded to a 0.96 unit increase in yield (**Figure 7f**), while a 1 unit increase in AGB resulted in a 0.23 unit rise in yield (**Figure 7c**). Previous studies (Yang et al., 2007) similarly demonstrated that the correlation between yield and HI is stronger than that between yield and biomass. Moreover, AGB was negatively correlated with HI; each 1 unit increase in AGB led to a 0.014 unit decrease in HI (**Figure 7i**). This inverse relationship suggests that increasing AGB alone may not enhance yield, as the concurrent decline in HI could offset potential gains. Huang et al. (2018) reported that the negative effects of reduced nitrogen application on grain yield can be compensated by increasing hill density, a mechanism attributed to enhanced biomass production. These findings align with the present study. The average yields of transplanted and direct-seeded rice in this study were 7.31 t ha^-1^ and 6.01 t ha^-1^, respectively, showing a significant difference. The corresponding above-ground biomass values were 13.58 t ha^-1^ and 13.42 t ha^-1^, respectively. However, the mean harvest index (HI) values were 0.54 for transplanted rice and 0.46 for direct-seeded rice, indicating that the conversion efficiency of above-ground biomass was relatively lower for direct-seeded rice. This difference in conversion efficiency was also observed across the treatments. Among the treatments, T1 achieved the highest yield and above-ground biomass values, at 7.27 t ha^-1^ and 16.4 t ha^-1^, respectively, with an HI of 0.46. In contrast, T4 had an above-ground biomass of 11.6 t ha^-1^, a yield of 6.01 t ha^-1^, and an HI of 0.52. The conversion efficiency of above-ground biomass to yield was lower in T1, indicating that yield accounted for a smaller of total biomass. Therefore, further increasing above-ground biomass did not significantly increase yield, emphasizing the importance of optimizing biomass allocation for improving utilization efficiency. Similarly, improving biomass conversion efficiency in direct-seeded rice is key to increasing yield. Compared to T1, T4 (38.80 cm spacing with 9 seedlings per hill) demonstrated higher conversion efficiency. Although total yield was lower, it exhibited more efficient biomass allocation. In conclusion, improving the allocation and conversion efficiency of above-ground biomass is crucial for maximizing rice yield.

The combined effects of number of seedlings per hill and plant spacing significantly affect the distribution of leaf and panicle biomass. As both factors increase, stem and leaf biomass allocation rise, while panicle biomass allocation decreases. The yield prediction model (**Figure 7a, b**) shows that biomass accumulation and allocation explain 31.32% of the yield variance. Specifically, each unit increase in leaf biomass (LB) results in a 0.044 unit increase in yield, while a unit increase in panicle biomass (PB) leads to a 0.09 unit increase. However, the interaction between LB and PB negatively affects yield, as increases in LB reduce PB's positive effect, lowering yield by 0.14 units. This trade-off in resource allocation between leaves and panicles intensifies internal competition, limiting yield improvement. Biomass allocation is dynamic, influenced by internal and external factors (Hiltbrunner et al., 2021; Xu et al., 2023). Optimal allocation theory suggests that plants prioritize biomass allocation to organs with the greatest resource constraints (McCarthy and Enquist, 2007; Chen and Weiner, 2024). Allometric growth theory indicates that organs exhibit distinct growth patterns, allowing flexibility in biomass allocation (Wang H. et al., 2024; Xiao et al., 2024). These trade-offs improve plant adaptability and competitiveness, contributing to ecosystem carbon cycling (Clarke et al., 2013; Freschet et al., 2018; Fang et al., 2023).

The simultaneous accumulation of organic carbon and biomass is crucial for rice yield formation. Increasing the number of seedlings per hill and plant spacing significantly reduces organic carbon accumulation in stems, leaves, and panicles. Stem carbon accumulation (SCA) negatively affects both yield and harvest index (HI), with each unit increase in SCA reducing yield by 0.286 units and HI by 0.211 units (**Figures 7a, g**). Similarly, the panicle carbon-nitrogen ratio (PCN) negatively impacts both yield and HI; each unit increase in PCN decreases yield by 0.024 and HI by 0.339. A high PCN indicates insufficient nitrogen supply, which may impair panicle development. However, when the stem carbon-nitrogen ratio (SCN) and leaf carbon-nitrogen ratio (LCN) balance with PCN, they positively influence grain yield. Both SCA and panicle carbon accumulation (PCA) positively contribute to above-ground biomass (AGB), but their combined effects reduce the individual contributions of each to AGB accumulation. This suggests that the regulatory role of AGB on yield and HI depends on the interaction between stem and panicle organic carbon accumulation. The carbon and nitrogen accumulation characteristics of hybrid and conventional rice exhibit similar patterns. In transplanted rice, GY9 showed a significant increase in SNA and LNA, which, however, resulted in a significant decrease in PNA. In contrast, hybrid rice varieties ZXY5 and YXY2 maintained high levels of panicle nitrogen accumulation, ultimately achieving the highest yield. In direct-seeded rice, the conventional rice variety ZGA had the lowest SNA and LNA but a significantly higher PNA. This suggests that in actual production, hybrid rice can maintain consistently high yields when transplanted, while the conventional rice variety ZGA is better suited for direct-seeded cultivation. Furthermore, YXY2 maximized tillering ability, effective panicle number, and leaf area index in transplanted rice, contributing to the construction of a high-yield structure.

During the vegetative and early reproductive stages, carbon from photosynthesis is either used for growth or stored as non-structural carbohydrates (NSC) in stems and leaf sheaths (Scofield et al., 2009). Approximately 30% of grain NSC comes from pre-heading accumulation, while about 70% is derived from post-heading assimilation, the primary carbon source for grain filling (CocK and YOSIIDA, 1972; Wada et al., 2017; Wakabayashi et al., 2022). Agricultural practices such as water management (Zang et al., 2024) and delaying nitrogen application until panicle initiation (Zhu et al., 2024) can enhance pre-heading NSC accumulation, improving grain filling. Excess stem carbohydrates help buffer the source-sink relationship and adapt to environmental fluctuations (Furze et al., 2018). In summary, increasing the number of seedlings per hill and plant spacing alters the source-sink relationship between the stem and panicle, affecting rice yield formation and conversion efficiency. While panicle biomass and carbon accumulation promote higher yields, excessive stem biomass and carbon accumulation disrupt this balance, ultimately reducing yield. For example, under 12.93 cm spacing with 1 seedlingsper hill (T1), both stem and panicle carbon accumulation increased significantly, but the harvest index (HI) reached its lowest value. This indicates that excessive carbon allocation to the stem impairs the source-sink balance and lowers conversion efficiency.

### 4.2 Pathways of rice yield formation and strategies for organic carbon allocation

In the structural equation model, biomass and organic carbon accumulation in stems, leaves, and panicles are key variables significantly influencing rice yield. These factors are integral to plant growth, nitrogen accumulation, and the carbon-nitrogen balance. Path analysis identifies two primary pathways through which the combined effects of seedling number per hill and plant spacing affect rice biomass accumulation and yield. In the first pathway, these variables directly enhance yield by increasing panicle biomass and organic carbon accumulation (PC2) and indirectly impact yield by boosting above-ground biomass and conversion efficiency, which in turn influence nitrogen accumulation and the carbon-nitrogen balance in the plant’s above-ground portions. The positive correlation between panicle biomass, organic carbon, nitrogen accumulation, and yield suggests that enhancing these factors can increase agricultural output. In the second pathway, combined effects of seedling number per hill and plant spacing directly affect plant growth and nitrogen accumulation by increasing stem, leaf, and panicle biomass along with organic carbon accumulation (PC1). This pathway also indirectly influences the carbon-nitrogen ratio through nitrogen accumulation. Unlike the first pathway, changes in stem, leaf, and panicle biomass, as well as organic carbon accumulation, affect yield indirectly through above-ground biomass and conversion efficiency. The standardized total effects of PC1 and PC2 on yield are 0.324 and 0.697, respectively, indicating that panicle biomass and organic carbon accumulation have a greater impact on yield. A crucial factor in determining yield is the availability and transport of assimilates, such as carbon and nitrogen, during grain development (Shao et al., 2021; Rosado-Souza et al., 2023; Fei et al., 2024). Limited nitrogen availability, particularly in maize, reduces nitrogen levels in the source (panicle and leaves), diminishes sink size and activity, and restricts carbon transport to the panicle, leading to lower yields (Ning et al., 2021). Pan et al. (2022) observed consistent source limitations during grain-filling under various fertilization treatments. A balanced source-sink relationship is essential for optimizing yield potential and crop productivity (Chang and Zhu, 2017). In this experiment, T4 had significantly lower panicle biomass than T1, limiting nitrogen and organic carbon accumulation and reducing sink capacity. Despite improved conversion efficiency, the potential for yield increases remained constrained. Biomass and organic carbon in stems and leaves constitute the source, while the panicle serves as the sink for biomass, organic carbon, and nitrogen. Increasing sink capacity positively influences nitrogen accumulation, indirectly affecting the carbon-nitrogen ratio and panicle biomass and organic carbon accumulation. Regulating biomass distribution between stems, leaves, and panicles creates a dynamic feedback loop essential for optimizing growth and yield. However, increasing the number of seedlings per hill and plant spacing gradually decrease biomass and carbon-nitrogen accumulation in stems and leaves, lowering panicle sink capacity.

The variations in resource allocation strategies by rice in response to different seedling numbers and plant spacing highlight trade-offs between traits, with increased seedling density and spacing constraining biomass accumulation due to source-sink limitations, while enhancing reliance on conversion efficiency to meet reproductive demands. These adaptations reflect rice's adjustment to its environment through coordinated trade-offs, supported by life-history allocation costs (Stearns, 1989), which explain trait variation. Plant resource allocation, such as defense versus growth (Giolai and Laine, 2024), often results in trade-offs that affect productivity, as seen in rice where correlations between stem and panicle traits negatively impact population productivity. However, rice mitigates these effects through compensatory mechanisms enhancing resource conversion efficiency. Plant plasticity, such as metabolic, structural, and physiological adaptations (Mieke et al., 2018; Peguero-Pina et al., 2020; Wang X. et al., 2024), helps alleviate stress and optimize resource uptake. These adaptations, such as adjustments in root and leaf traits under nitrogen and light stress (Freschet et al., 2018), improve productivity. In intercropping systems, such trade-offs, like those between soybean and maize, enhance nitrogen utilization (Yang et al., 2023), contributing to functional diversity and ecosystem resilience (Blesh, 2018). Breeding practices have shifted rice traits, affecting crop performance under high-resource conditions, and future efforts should focus on optimizing photosynthetic enzyme efficiency (Xiong and Flexas, 2018). In agricultural settings, treatments like T1 facilitate higher yields through effective resource allocation and trait trade-offs, with further improvements in material conversion efficiency critical for maximizing yield potential.

## 5 Conclusion

Under identical seedling conditions, variations in the number of seedlings per hill and plant spacing significantly influence rice yield. The highest yields in both transplanting and direct seeding methods occur with single seedlings and narrow row spacing (12.93 cm spacing with 1 seedling per hill). These effects primarily result from trade-offs between stem and panicle traits and shifts in resource allocation strategies. Accumulating and allocating above-ground biomass is crucial for achieving high yields with single seedlings and narrow spacing. As seedling number and spacing increase, biomass allocation to stems and leaves decreases, while allocation to panicles increases, improving material conversion efficiency. Above-ground biomass plays a vital role in regulating yield. As seedling number and spacing increase, panicle biomass and organic carbon accumulation rise, positively impacting yield. However, simultaneous increases in stem and panicle biomass can disrupt the source-sink balance, negatively affecting yield. The regulatory pathways through which seedling number and spacing affect yield can be categorized into two mechanisms: a direct positive effect on panicle biomass and organic carbon accumulation, and changes in overall biomass and carbon accumulation. Notably, above-ground biomass and material conversion efficiency exert a significant positive indirect effect on yield. Consequently, the trade-offs in single seedlings and narrow spacing (12.93 cm spacing with 1 seedling per hill) facilitate synchronized increases in stem, leaf, and grain biomass, enabling higher yields even under conditions of low conversion efficiency.

## Data Availability

The datasets presented in this study can be found in online repositories. The names of the repository/repositories and accession number(s) can be found in the article/**Supplementary Material**.
