# Supplementary material for: Interactive effects of seedling number per hill and plant spacing on source-sink dynamics and yield formation in rice
Source: Front Plant Sci. 2025 Nov 24;16:1707882. doi: 10.3389/fpls.2025.1707882 (PMC12682863; doi:10.3389/fpls.2025.1707882)
Supplement: Supplementary file 1 [file DataSheet1.pdf]

Article title：Planting single rice seedlings at close intervals enhances yields by promoting biomass accumulation and mitigating low conversion efficiency.

Author: Xiaoyan WU, Dongjie Xie, Anjie Xu, Pengli Yuan, Jiada Huang, Ligeng Jiang

The following Supporting Information is available for this article:

Figure S1 Temperature and precipitation during the rice growing season in Nanning and Yulin in 2020-2021.

Figure S2 Effects of rice varieties and treatments on tillers and leaf area index.

Figure S3 Effects of rice varieties and treatments on number of effective panicles and specific leaf area.

Figure S4 Effects of variety and treatment on carbon-to-nitrogen in stem, leaf, panicle.

Figure S5 Principal component analysis of stem, leaf and ear biomass and organic carbon accumulation.

Figure S6 Path analysis showing the effects of rice growth, biomass accumulation, nitrogen accumulation, organic carbon accumulation, carbon-nitrogen ratio, total biomass accumulation, and material transport efficiency on yield.

Figure S7 Linear regression models of stem-sheath carbon-nitrogen ratio (SCN), leaf carbon-nitrogen ratio (LCN), panicle carbon-nitrogen ratio (PCN) and PC1 and PC2.

Table S1 Basic physical and chemical properties of soil in Nanning and Yulin.

Table S2 Experimental plot area and plant spacing settings.

Table S3 Statistical summary of the linear mixed effects model of the effects of variety and treatment (plant spacing and seedling number per hill configuration) on rice yield and aboveground traits.

Table S4 Summary of post hoc test statistics for the effects of variety and treatment (plant spacing and seedling number per hill configuration) on rice yield and aboveground traits.

Table S5 The best models for predicting yield, AGB, and HI based on AICc selection.

Table S6 Names and abbreviations of above-ground traits of rice plants as used in this study.

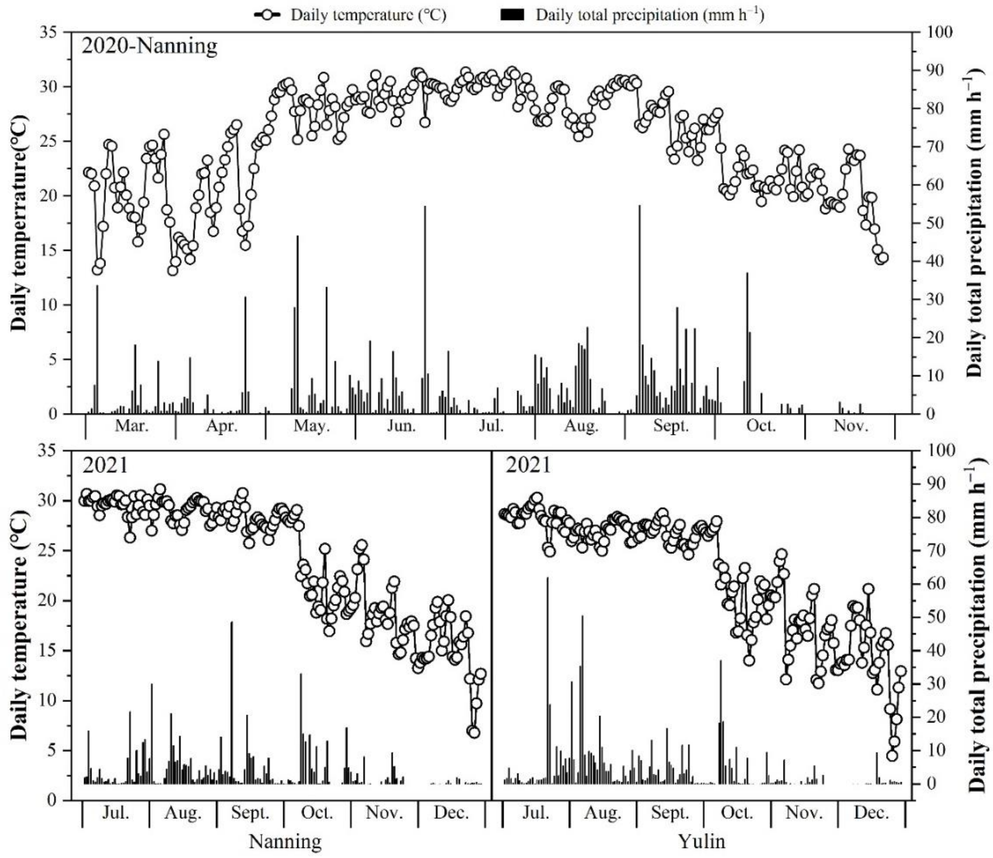

Figure S1 Temperature and precipitation during the rice growing season in Nanning and Yulin in 2020-2021.

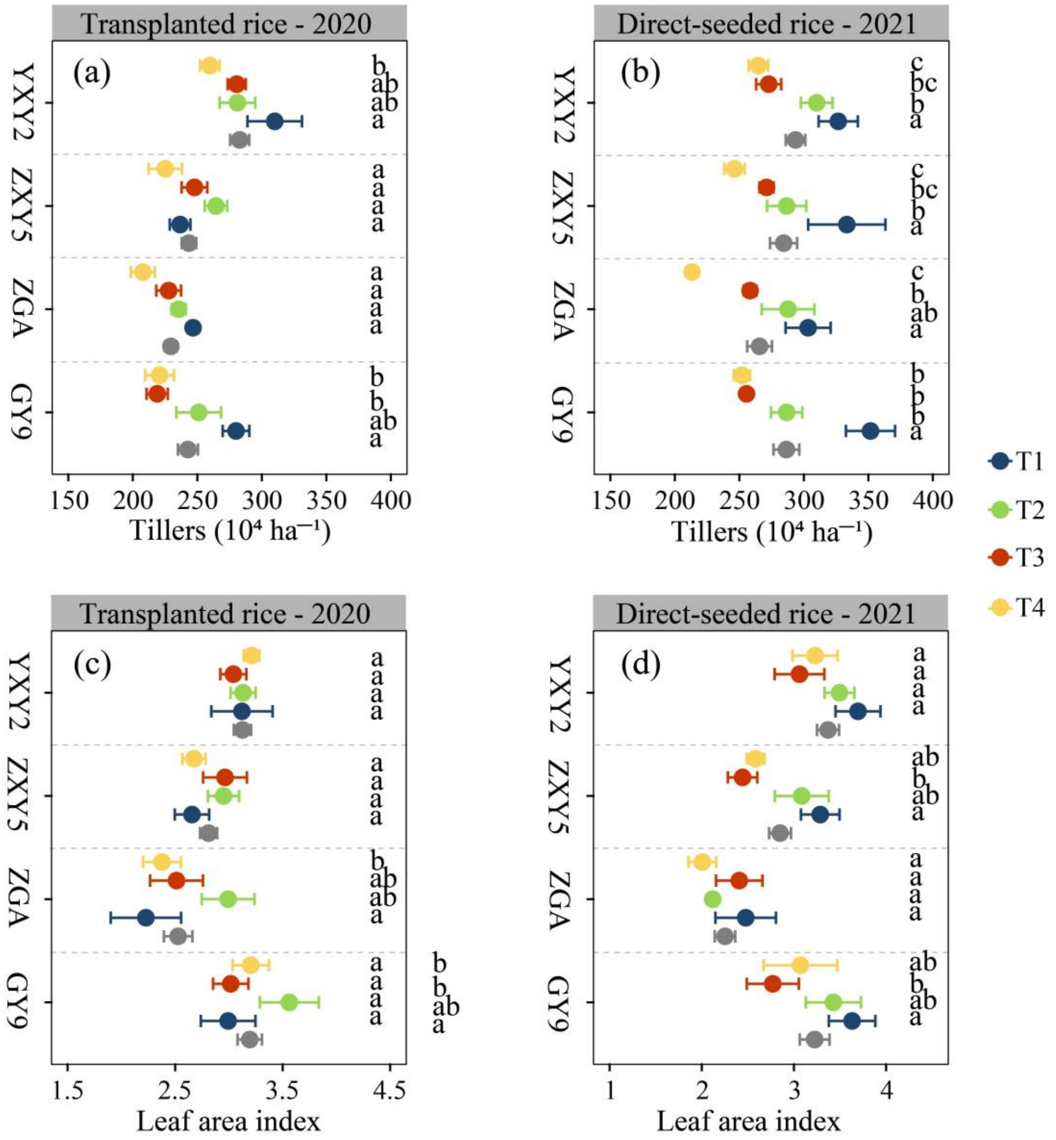

Figure S2 Effects of rice varieties and treatments on tillers and leaf area index. Panels (a) and (c) demonstrate the effects of treatments on the tillers and LAI in transplanted rice. Panels (b) and (d) highlight these effects in direct-seeded rice. GY9, Guiyu 9; ZGA, Zhenguiai; ZXY5, Zhuangxiangyoubaijin 5; YXY2, Yexiangyou 2. T1 = 12.93 cm spacing, 1 seedling per hill; T2 = 22.33 cm spacing, 3 seedlings per hill; T3 = 31.67 cm spacing, 6 seedlings per hill; T4 = 38.80 cm spacing, 9 seedlings per hill. Gray dots represent the mean of different varieties. Lowercase letters denote significant differences among treatments ($p < 0.05$).

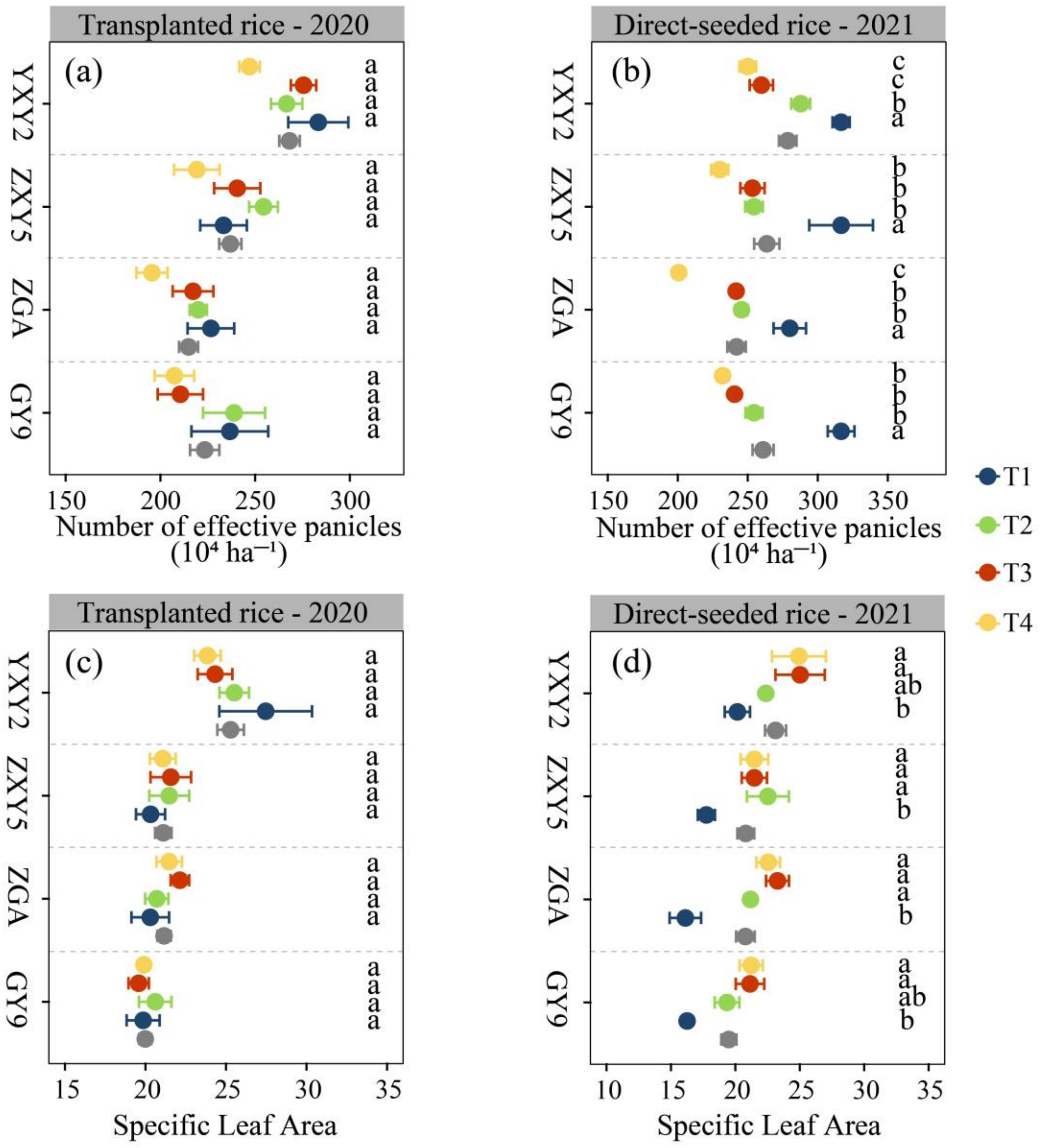

Figure S3 Effects of rice varieties and treatments on number of effective panicles and specific leaf area. Panels (a) and (c) demonstrate the effects of treatments on the number of effective panicles and specific leaf area in transplanted rice. Panels (b) and (d) highlight these effects in direct-seeded rice. GY9, Guiyu 9; ZGA, Zhenguiai; ZXY5, Zhuangxiangyoubaijin 5; YXY2, Yexiangyou 2. T1 = 12.93 cm spacing, 1 seedling per hill; T2 = 22.33 cm spacing, 3 seedlings per hill; T3 = 31.67 cm spacing, 6 seedlings per hill; T4 = 38.80 cm spacing, 9 seedlings per hill. Gray dots represent the mean of different varieties. Lowercase letters denote significant differences among treatments ($p < 0.05$).

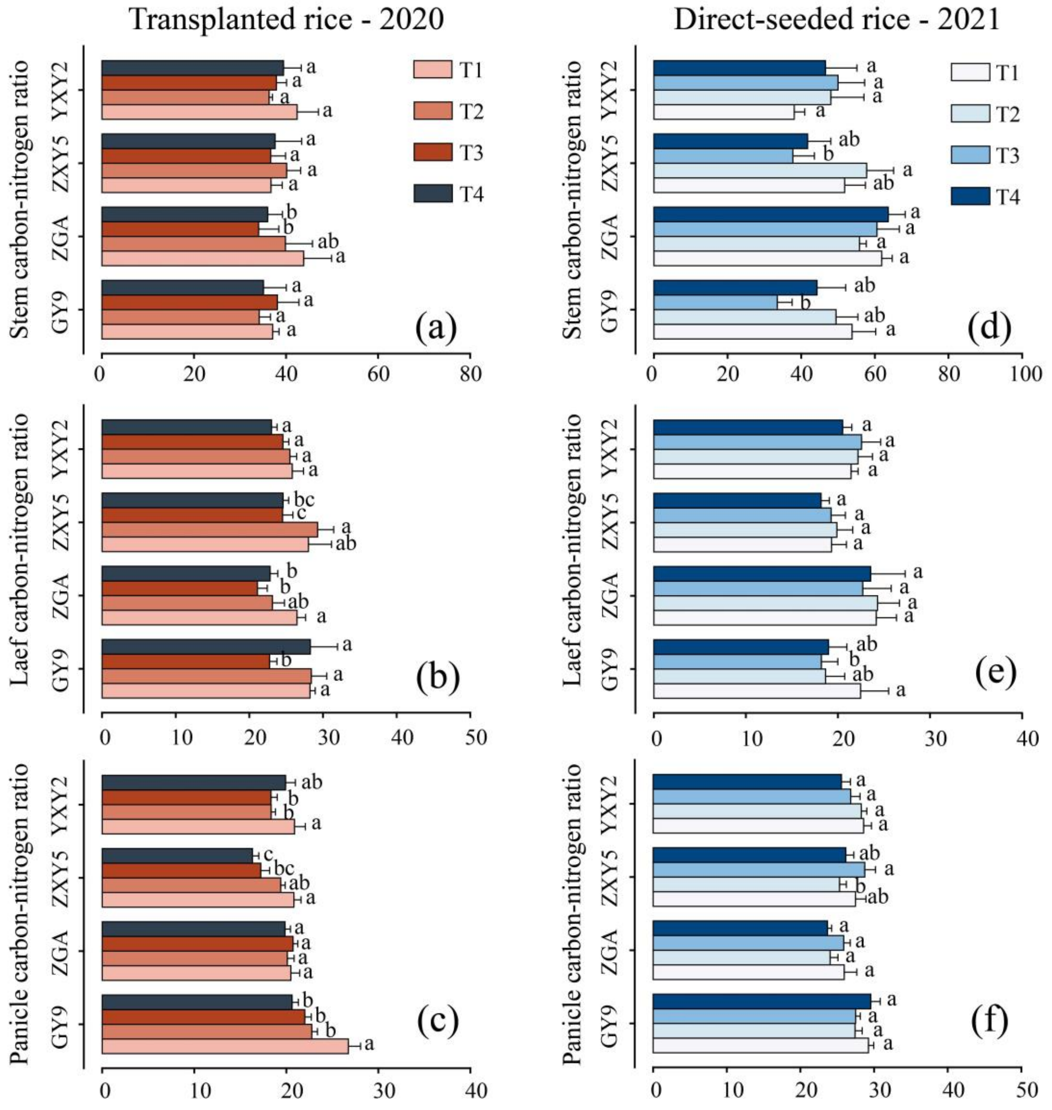

Figure S4 Effects of variety and treatment on carbon-to-nitrogen in stem, leaf, panicle. GY9, Guiyu 9; ZGA, Zhenguiai; ZXY5, Zhuangxiangyoubaijin 5; YXY2, Yexiangyou 2. T1 = 12.93 cm spacing with 1 seedling per hill; T2 = 22.33 cm spacing with 3 seedlings per hill; T3 = 31.67 cm spacing with 6 seedlings per hill; T4 = 38.80 cm spacing with 9 seedlings per hill. Lowercase letters denote significant differences among treatments ($p < 0.05$).

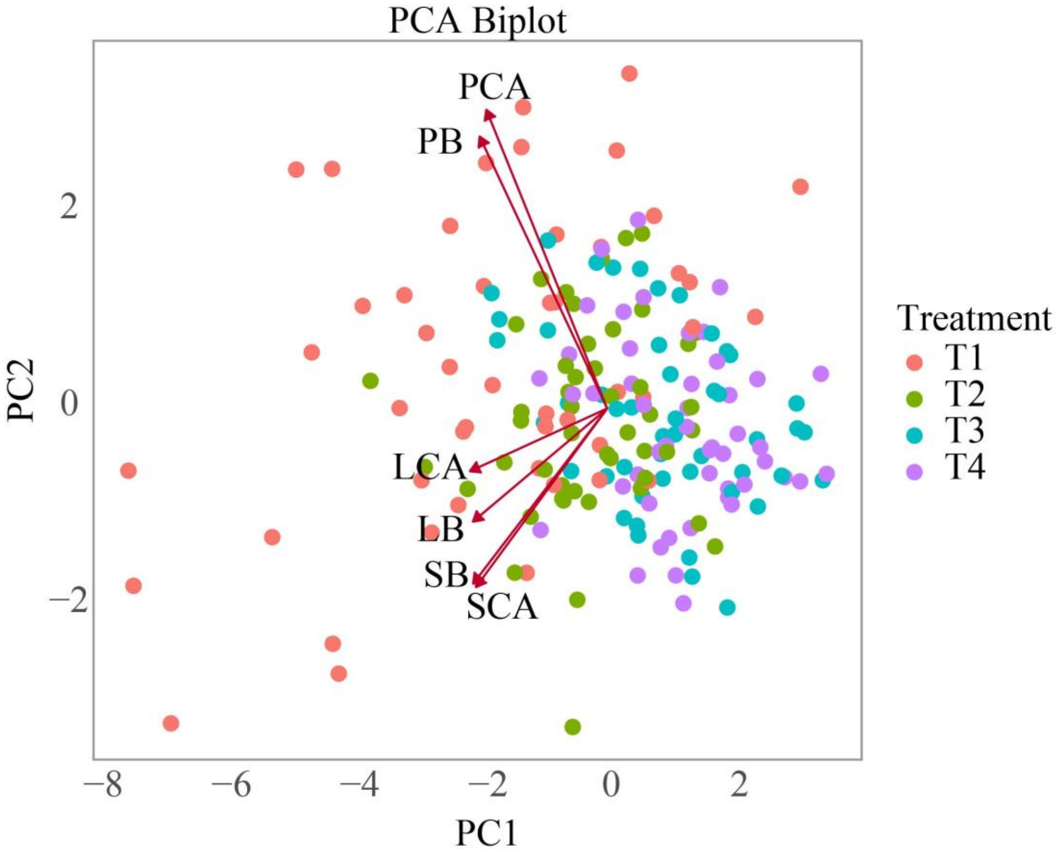

Figure S5 Principal component analysis of stem, leaf and ear biomass and organic carbon accumulation. Note: SB, SCA: stem biomass accumulation and organic carbon accumulation; LB, LCA: leaf biomass accumulation and organic carbon accumulation; PB, PCA: panicle biomass accumulation and organic carbon accumulation. Among the principal components, PC1 indicates that rice accumulates biomass and organic carbon in stems, leaves, and panicles simultaneously, PC2 indicates that rice tends to accumulate biomass and organic carbon in the panicle. T1 = 12.93 cm spacing, 1 seedling per hill; T2 = 22.33 cm spacing, 3 seedlings per hill; T3 = 31.67 cm spacing, 6 seedlings per hill; T4 = 38.80 cm spacing, 9 seedlings per hill.

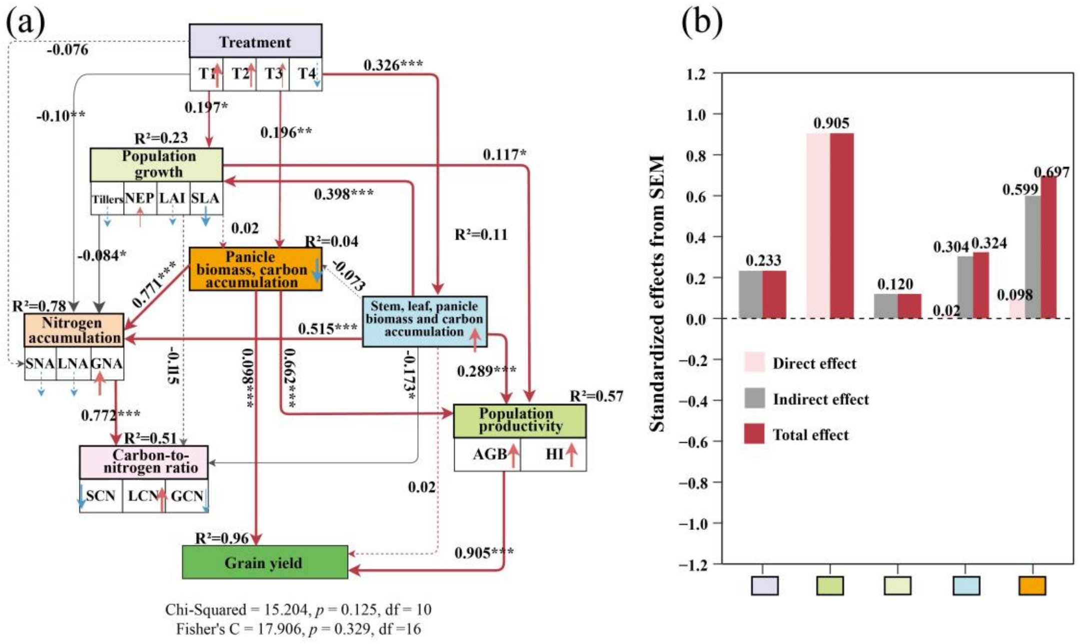

Figure S6 Path analysis showing the effects of rice growth, biomass accumulation, nitrogen accumulation, organic carbon accumulation, carbon-nitrogen ratio, total biomass accumulation, and material transport efficiency on yield. ‘Panicle biomass, carbon accumulation’ and ‘Stem, leaf panicle biomass and carbon accumulation’ represent PC2 and PC1, respectively, from the principal component analysis of stem, leaf, grain biomass, and organic carbon accumulation (Figure S5). T1 = 12.93 cm spacing with 1 seedling per hill; T2 = 22.33 cm spacing with 3 seedlings per hill; T3 = 31.67 cm spacing with 6 seedlings per hill; T4 = 38.80 cm spacing with 9 seedlings per hill. Population growth is a composite variable consisting of tiller number, number of effective panicles, leaf area index, and specific leaf area. Nitrogen accumulation is a composite variable consisting of stem nitrogen accumulation, leaf nitrogen accumulation, and panicle nitrogen accumulation. Carbon-to-nitrogen Ratio is a composite variable consisting of stem carbon-nitrogen ratio, leaf carbon-nitrogen ratio, and panicle carbon-nitrogen ratio. Population productivity is a composite variable consisting of above-ground biomass and harvest index. Dashed arrows indicate statistically insignificant relationships between variables. Gray solid arrows and red solid arrows indicate significant negative and positive relationships, respectively. ***, $p < 0.001$; **, $p < 0.01$; *, $p < 0.05$.

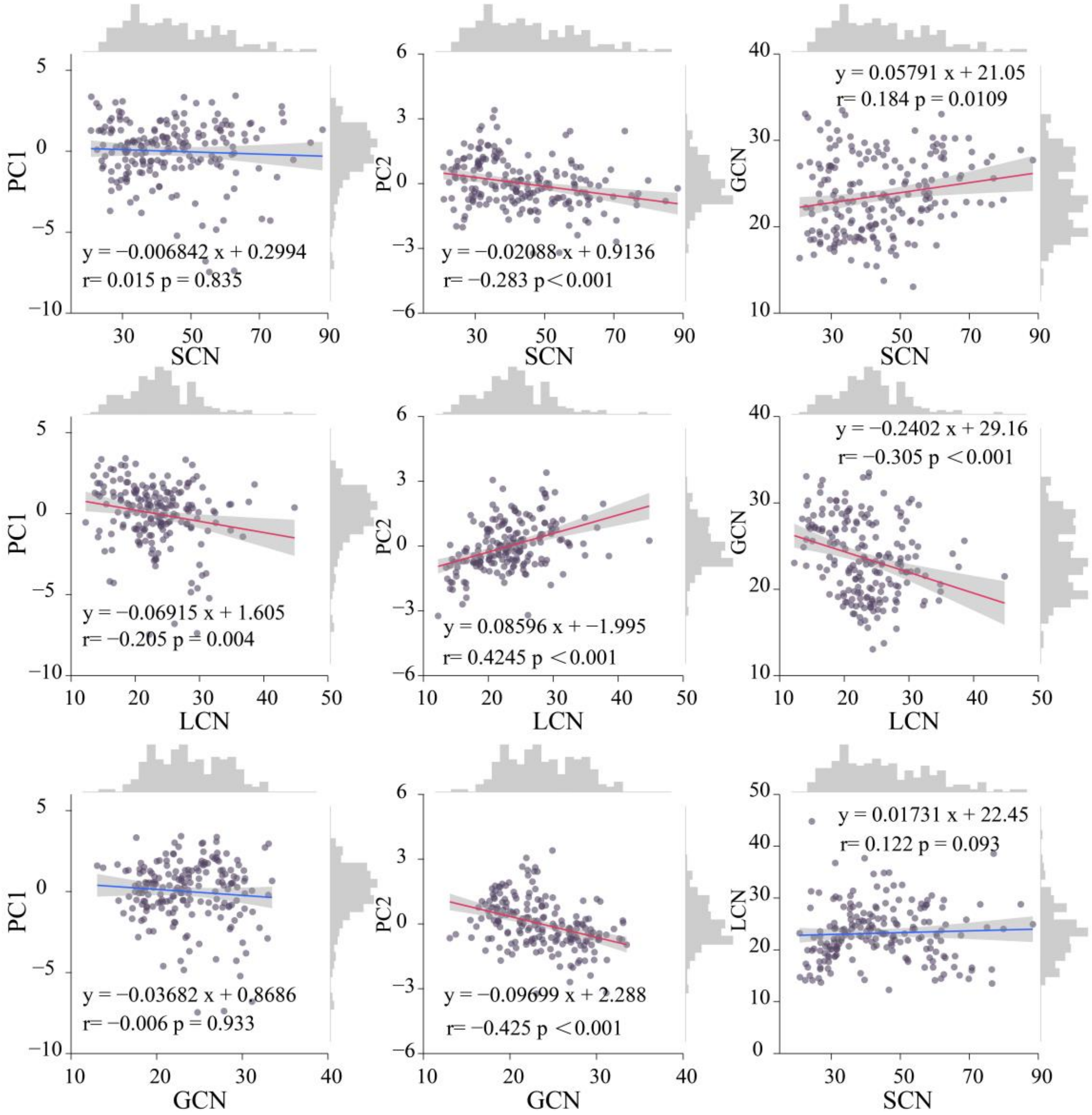

Figure S7 Linear regression models of stem carbon-nitrogen ratio (SCN), leaf carbon-nitrogen ratio (LCN), panicle carbon-nitrogen ratio (PCN) and PC1 and PC2.

Table S1 Basic physical and chemical properties of soil

Site	pH	Organic carbon（g kg^{-1}）	Total N (g kg^{-1})	Total P (g kg^{-1})	Total K (g kg^{-1})
Nanning	6.5	19.1	1.513	1.34	6.7
Yulin	5.17	16.13	0.88	1.03	15.86

Table S2 Experimental plot area and plant spacing settings

Treatment	Seedling number ($hill^{-1}$)	Plot area (m^2)	Basic density (m^{-2})	Total seedling number ($plot^{-1}$)	Row/Line number	Row spacing (cm)
T1	1	3.75(1.94×1.94)	60	225	15×15	12.93×12.93
T2	3	4.05(2.01×2.01)	60	243	9×9	22.33×22.33
T3	6	3.60(1.90×1.90)	60	216	6×6	31.67×31.67
T4	9	3.75(1.94×1.94)	60	225	5×5	38.80×38.80

T1, 12.93 cm spacing with 1 seedling per hill. T2, 22.33 cm spacing with 3 seedlings per hill. T3, 31.67 cm spacing with 6 seedlings per hill. T4, 38.80 cm spacing with 9 seedlings per hill.

Table S3 Statistical summary of the linear mixed effects model of the effects of variety and treatment (plant spacing and seedling number per hill configuration) on rice yield and aboveground traits.

Response variable	Best model random effects structure		Fixed effects	Standardized estimate (β)	Standard error	df	P value	Significance	Conditional R^2
Yield	random = (1 | Planting) +(1 | Site) + (1 | Season)		Variety	0.54	0.210	184.96	0.010	*	0.93
	σ^2	0.42	Treatment	-1.27	0.210	184.96	0.000	***	
	$\tau00_{\text{Planting}}$	0.37	Variety × Treatment	0.02	0.337	184.96	0.949		
	$\tau00_{\text{Site}}$	1.36							
	$\tau00_{\text{Season}}$	3.55							
	ICC	0.93							
	N_{Planting}	2.00							
	N_{Site}	2.00							
	N_{Season}	2.00							
Aboveground biomass	random = (1 | Planting) +(1 | Site) + (1 | Season)		Variety	-0.74	0.636	184.77	0.244		0.692
	σ^2	3.85	Treatment	-5.62	0.636	184.77	0.000	***	
	$\tau00_{\text{Planting}}$	1.41	Variety × Treatment	1.54	1.020	184.77	0.134		
	$\tau00_{\text{Site}}$	3.14							
	$\tau00_{\text{Season}}$	0.79							
	ICC	0.58							
	N_{Planting}	2							
	N_{Site}	2							
	N_{Season}	2							
Harvest index	random = (1 | Planting) +(1 | Site) + (1 | Season)		Variety	0.05	0.025	186.04	0.071	.	NA
	σ^2	0.01	Treatment	0.08	0.025	186.04	0.002	**	
	$\tau00_{\text{Planting}}$	0	Variety × Treatment	-0.03	0.040	186.04	0.519		

Response variable	Best model random effects structure		Fixed effects	Standardized estimate (β)	Standard error	df	P value	Significance	Conditional R^2
	$\tau00_{Site}$	0							
	$\tau00_{Season}$	0.01							
	$N_{Planting}$	2							
	N_{Site}	2							
	N_{Season}	2							
Tillers	random = (1 | Planting) +(1 | Site) + (1 | Season)		Variety	12.82	10.954	185.45	0.2433		NA
	σ^2	1142.84	Treatment	-75.82	10.954	185.45	7.08E-11	***	
	$\tau00_{Planting}$	531.94	Variety × Treatment	26.68	17.566	185.45	0.1306		
	$\tau00_{Site}$	0.5							
	$\tau00_{Season}$	0							
	$N_{Planting}$	2							
	N_{Site}	2							
	N_{Season}	2							
Number of effective panicles	random = (1 | Planting) +(1 | Site) + (1 | Season)		Variety	30.39	9.233	184.59	0.00119	**	0.599
	σ^2	811.82	Treatment	-55.49	9.233	184.59	9.68E-09	***	
	$\tau00_{Planting}$	573.06	Variety × Treatment	8.46	14.805	184.59	0.56829		
	$\tau00_{Site}$	29.81							
	$\tau00_{Season}$	71.77							
	ICC	0.45							
	$N_{Planting}$	2							
	N_{Site}	2							
	N_{Season}	2							
Leaf area index	random = (1 | Planting) +(1 | Site) + (1 | Season)		Variety	0.16	0.21383	187	0.453		0.055
	σ^2	0.44	Treatment	-0.30	0.21383	187	0.168		

Response variable	Best model random effects structure		Fixed effects	Standardized estimate (β)	Standard error	df	P value	Significance	Conditional R^2
	$\tau00_{Planting}$	0	Variety × Treatment	0.01	0.3429	187	0.972		
	$\tau00_{Site}$	0.01							
	$\tau00_{Season}$	0							
	ICC	0.02							
	$N_{Planting}$	2							
	N_{Site}	2							
	N_{Season}	2							
Specific leaf area	random = (1 | Planting) +(1 | Site) + (1 | Season)		Variety	5.06	0.8936	184.84	5.58E-08	***	0.664
	σ^2	7.61	Treatment	3.28	0.8936	184.84	0.00031	***	
	$\tau00_{Planting}$	5.39	Variety × Treatment	-2.09	1.433	184.84	0.1459		
	$\tau00_{Site}$	1.99							
	$\tau00_{Season}$	4.62							
	ICC	0.61							
	$N_{Planting}$	2							
	N_{Site}	2							
	N_{Season}	2							
Stem biomass	random = (1 | Planting) +(1 | Site) + (1 | Season)		Variety	-0.81	0.3121	185.07	0.0101	*	0.527
	σ^2	0.93	Treatment	-1.83	0.3121	185.07	1.99E-08	***	
	$\tau00_{Planting}$	0.49	Variety × Treatment	0.73	0.5006	185.07	0.149		
	$\tau00_{Site}$	0.17							
	$\tau00_{Season}$	0.03							
	ICC	0.43							
	$N_{Planting}$	2							
	N_{Site}	2							

Response variable	Best model random effects structure		Fixed effects	Standardized estimate (β)	Standard error	df	P value	Significance	Conditional R^2
	N_{Season}	2							
Leaf biomass	random = (1 | Planting) +(1 | Site) + (1 | Season)		Variety	-0.19	0.1062	185.14	0.07446	.	0.232
	σ^2	0.11	Treatment	-0.41	0.1062	185.14	0.00016	***	
	$\tau 00_{Planting}$	0	Variety × Treatment	0.13	0.1704	185.14	0.45814		
	$\tau 00_{Site}$	0							
	$\tau 00_{Season}$	0.01							
	ICC	0.11							
	$N_{Planting}$	2							
	N_{Site}	2							
	N_{Season}	2							
Panicles biomass	random = (1 | Planting) +(1 | Site) + (1 | Season)		Variety	0.26	0.369	184.79	0.484		0.771
	σ^2	1.30	Treatment	-3.38	0.369	184.79	<0.001	***	
	$\tau 00_{Planting}$	0.20	Variety × Treatment	0.68	0.5918	184.79	0.2495		
	$\tau 00_{Site}$	1.78							
	$\tau 00_{Season}$	1.04							
	ICC	0.70							
	$N_{Planting}$	2.00							
	N_{Site}	2.00							
	N_{Season}	2.00							
Stem mass fraction	random = (1 | Planting) +(1 | Site) + (1 | Season)		Variety	-0.03	0.011669	185.21	0.00902	**	0.680
	σ^2	0	Treatment	0.03	0.011669	185.21	0.00604	**	
	$\tau 00_{Planting}$	0	Variety × Treatment	-0.01	0.018712	185.21	0.7795		
	$\tau 00_{Site}$	0							
	$\tau 00_{Season}$	0							

Response variable	Best model random effects structure		Fixed effects	Standardized estimate (β)	Standard error	df	P value	Significance	Conditional R^2
	ICC	0.66							
	$N_{Planting}$	2							
	N_{Site}	2							
	N_{Season}	2							
Leaf mass fraction	random = (1 | Planting) +(1 | Site) + (1 | Season)		Variety	-0.01	0.006799	185.66	0.2808		NA
	σ^2	0	Treatment	0.01	0.006799	185.66	0.032	*	
	$\tau00_{Planting}$	0	Variety × Treatment	-0.01	0.010903	185.66	0.622		
	$\tau00_{Site}$	0							
	$\tau00_{Season}$	0							
	$N_{Planting}$	2							
	N_{Site}	2							
	N_{Season}	2							
Panicle mass fraction	random = (1 | Planting) +(1 | Site) + (1 | Season)		Variety	0.04	0.01314	185.24	0.00415	**	0.686
	σ^2	0	Treatment	-0.05	0.01314	185.24	0.00043	***	
	$\tau00_{Planting}$	0	Variety × Treatment	0.01	0.02108	185.24	0.61455		
	$\tau00_{Site}$	0							
	$\tau00_{Season}$	0							
	ICC	0.65							
	$N_{Planting}$	2							
	N_{Site}	2							
	N_{Season}	2							
Stem nitrogen accumulation	random = (1 | planting) +(1 | site) + (1 | season)		Variety	-2.86	4.357	185.9	0.5129		0.394
	σ^2	180.82	Treatment	-4.86	4.357	185.9	0.2662		

Response variable	Best model random effects structure		Fixed effects	Standardized estimate (β)	Standard error	df	P value	Significance	Conditional R^2
	$\tau00_{planting}$	0	Variety × Treatment	-5.37	6.987	185.9	0.4432		
	$\tau00_{site}$	13.83							
	$\tau00_{season}$	90.95							
	ICC	0.37							
	$N_{planting}$	2							
	N_{site}	2							
	N_{season}	2							
Leaf nitrogen accumulation	random = (1 | planting) +(1 | site) + (1 | season)		Variety	-2.31	2.6029	184.68	0.376		0.453
	σ^2	64.53	Treatment	-4.28	2.6029	184.68	0.102		
	$\tau00_{planting}$	3.11	Variety × Treatment	-0.18	4.174	184.68	0.967		
	$\tau00_{site}$	37.88							
	$\tau00_{season}$	8.99							
	ICC	0.44							
	$N_{planting}$	2							
	N_{site}	2							
	N_{season}	2							
Panicle nitrogen accumulation	random = (1 | planting) +(1 | site) + (1 | season)		Variety	25.94	9.259	185.13	0.00564	**	0.768
	σ2	816.53	Treatment	-47.28	9.259	185.13	8.12E-07	***	
	$\tau00_{planting}$	229.05	Variety × Treatment	6.41	14.848	185.13	0.66642		
	$\tau00_{site}$	1008.97							
	$\tau00_{season}$	1071.21							
	ICC	0.74							
	$N_{planting}$	2							
	N_{site}	2							

Response variable	Best model random effects structure		Fixed effects	Standardized estimate (β)	Standard error	df	P value	Significance	Conditional R^2
	N_{season}	2							
Stem organic carbon accumulation	random = (1 | Planting) +(1 | Site) + (1 | Season)		Variety	-391.56	131.781	185.01	0.00336	**	0.545
	σ^2	165392.98	Treatment	-754.45	131.781	185.01	4.11E-08	***	
	$\tau00_{Planting}$	52322.84	Variety × Treatment	295.49	211.32	185.01	0.1637		
	$\tau00_{Site}$	52442.05							
	$\tau00_{Season}$	31846.98							
	ICC	0.45							
	$N_{Planting}$	2							
	N_{Site}	2							
	N_{Season}	2							
Leaf organic carbon accumulation	random = (1 | Planting) +(1 | Site) + (1 | Season)		Variety	-56.41	44.166	187	0.20313		NA
	σ^2	18577.82	Treatment	-167.69	44.166	187	0.0002	***	
	$\tau00_{Planting}$	0	Variety × Treatment	47.26	70.824	187	0.50539		
	$\tau00_{Site}$	0							
	$\tau00_{Season}$	2292.4							
	$N_{Planting}$	2							
	N_{Site}	2							
	N_{Season}	2							
Panicle organic carbon accumulation	random = (1 | Planting) +(1 | Site) + (1 | Season)		Variety	133.38	167.54	184.87	0.427		0.831
	σ^2	267330.68	Treatment	-1482.23	167.54	184.87	7.14E-16	***	
	$\tau00_{Planting}$	55896.97	Variety × Treatment	267.66	268.662	184.87	0.3204		
	$\tau00_{Site}$	429853.28							
	$\tau00_{Season}$	562858.66							
	ICC	0.8							

Response variable	Best model random effects structure		Fixed effects	Standardized estimate (β)	Standard error	df	P value	Significance	Conditional R^2
	$N_{Planting}$	2							
	N_{Site}	2							
	N_{Season}	2							
Stem carbon-nitrogen ratio	random = (1 | Planting) +(1 | Site) + (1 | Season)		Variety	-4.37	3.734	184.96	0.2431		0.625
	σ^2	132.79	Treatment	-7.41	3.734	184.96	0.0486	*	
	$\tau00_{Planting}$	45.68	Variety × Treatment	7.58	5.988	184.96	0.2073		
	$\tau00_{Site}$	57.38							
	$\tau00_{Season}$	114.86							
	ICC	0.62							
	$N_{Planting}$	2							
	N_{Site}	2							
	N_{Season}	2							
Leaf carbon-nitrogen ratio	random = (1 | Planting) +(1 | Site) + (1 | Season)		Variety	-0.50	1.3172	182.47	0.7072		0.652
	σ^2	16.52	Treatment	-2.65	1.3172	182.47	0.0458	*	
	$\tau00_{Planting}$	0.31	Variety × Treatment	0.51	2.1121	182.47	0.8089		
	$\tau00_{Site}$	29.47							
	$\tau00_{Season}$	0.34							
	ICC	0.65							
	$N_{Planting}$	2							
	N_{Site}	2							
	N_{Season}	2							
Panicle carbon-nitrogen ratio	random = (1 | Planting) +(1 | Site) + (1 | Season)		Variety	-2.21	0.8903	184.93	0.0138	*	0.739
	σ^2	7.55	Treatment	-2.11	0.8903	184.93	0.0186	*	
	$\tau00_{Planting}$	18.95	Variety × Treatment	0.18	1.4276	184.93	0.8986		

Response variable	Best model random effects structure		Fixed effects	Standardized estimate (β)	Standard error	df	P value	Significance	Conditional R^2
	$\tau 00_{Site}$	1.05							
	$\tau 00_{Season}$	0.19							
	ICC	0.73							
	$N_{Planting}$	2							
	N_{Site}	2							
	N_{Season}	2							

In the tested model results, the fixed effects and random effects of the model are shown. The table also gives the degrees of freedom, Conditional R^2, and *p* values for each variable and possible interactions. Significance codes: 0 ‘***’, 0.001 ‘**’, 0.01 ‘*’, 0.05 ‘.’, 0.1 ‘ ’.

Table S4 Summary of post hoc test statistics for the effects of variety and treatment (plant spacing and seedling number per hill configuration) on rice yield and aboveground traits.

Planting method	Season	Variety	Treatment	Grain yield (t ha^{-1})	AGB (t ha^{-1})	HI (%)	Tillers (10^4 ha^{-1})	NEP (10^4 ha^{-1})	LAI	SLA	SB (t ha^{-1})	LB (t ha^{-1})	PB (t ha^{-1})	SMF	LMF	PMF	SNA (kg ha^{-1})	LNA (kg ha^{-1})	PNA (kg ha^{-1})	SCA (kg ha^{-1})	LCA (kg ha^{-1})	PCA (kg ha^{-1})	SCN	LCN	PCN
**Transplanted rice**	**Early season**	**GY9**	**T1**	8.95 a	17.20 a	0.53 a	280.0 a	273.33 a	3.40 a	18.35 a	5.53 a	1.85 a	9.82 a	0.32 a	0.11 a	0.57 a	63.64 a	26.32 ab	161.24 a	2218.31 a	783.01 a	4789.03 a	35.30 a	29.70 b	29.64 a
			**T2**	8.55 a	15.06 ab	0.57 a	235.56 a	222.22 a	3.51 a	18.89 a	4.88 a	1.86 a	8.32 ab	0.32 a	0.12 a	0.55 ab	65.35 a	25.04 ab	174.57 a	1892.59 a	814.17 a	4168.19 ab	29.39 ab	32.55 ab	23.87 b
			**T3**	7.56 ab	12.68 b	0.60 a	234.44 a	231.11 a	3.09 a	18.41 a	4.72 a	1.67 a	6.29 c	0.37 a	0.13 a	0.49 b	63.50 a	33.13 a	144.10 a	1839.38 a	687.57 a	3112.90 c	29.28 ab	20.82 c	21.55 b
			**T4**	7.09 b	13.16 b	0.54 a	234.81 a	226.67 a	3.52 a	20.28 a	4.26 a	1.74 a	7.17 bc	0.32 a	0.13 a	0.54 ab	68.10 a	22.60 b	169.93 a	1643.37 a	749.73 a	3492.73 bc	24.14 b	34.73 a	20.70 b
		**ZGA**	**T1**	8.97 a	15.54 a	0.58 a	253.33 a	246.67 a	2.81 a	19.09 a	4.54 a	1.51 a	9.48 a	0.29 a	0.10 a	0.61 a	59.61 a	28.55 a	248.43 a	1804.93 a	680.57 a	4591.29 a	30.46 a	23.88 a	18.45 a
			**T2**	8.14 ab	11.97 b	0.68 a	237.78 a	224.44 a	2.47 a	19.70 a	3.89 a	1.25 a	6.83 b	0.33 a	0.10 a	0.57 a	57.46 a	28.24 a	176.05 b	1538.42 a	553.01 a	3308.21 b	27.13 a	19.60 ab	18.79 a
			**T3**	8.23 ab	13.29 ab	0.62 a	235.56 a	225.56 a	3.01 a	21.04 a	4.10 a	1.43 a	7.76 ab	0.31 a	0.11 a	0.58 a	66.25 a	34.45 a	188.31 b	1615.15 a	629.78 a	3833.67 ab	24.54 a	18.30 b	20.39 a
			**T4**	7.47 b	11.59 b	0.65 a	202.22 a	190.37 a	2.52 a	19.75 a	3.51 a	1.28 a	6.80 b	0.30 a	0.11 a	0.59 a	49.52 a	27.07 a	179.38 b	1435.59 a	573.42 a	3369.71 b	29.30 a	21.14 ab	18.76 a
		**ZXY2**	**T1**	9.51 a	16.70 a	0.57 a	233.33 a	226.67 a	2.73 a	18.75 a	4.67 a	1.46 a	10.57 a	0.28 a	0.09 a	0.63 a	61.54 a	30.75 a	233.03 a	1930.16 a	648.49 a	5081.88 a	31.74 a	21.20 a	21.83 a
			**T2**	8.92 ab	14.01 ab	0.64 a	248.89 a	244.44 a	2.84 a	19.09 a	4.66 a	1.48 a	7.87 bc	0.33 a	0.11 a	0.56 b	55.57 a	25.90 a	209.42 a	1859.65 a	633.88 a	3992.53 bc	33.59 a	24.49 a	19.13 ab

		**T3**	8.65 ab	16.13 a	0.54 a	268.89 a	265.56 a	3.16 a	19.24 a	5.44 a	1.63 a	9.06 ab	0.34 a	0.10 a	0.56 b	71.47 a	33.28 a	245.27 a	2107.80 a	714.31 a	4399.03 ab	29.64 a	21.52 a	17.95 b
		**T4**	7.95 b	12.67 b	0.63 a	237.04 a	231.11 a	2.78 a	19.70 a	4.05 a	1.42 a	7.21 c	0.32 a	0.11 a	0.57 ab	63.86 a	26.54 a	207.41 a	1574.89 a	620.84 a	3497.16 c	25.09 a	23.38 a	16.87 b
	**YXY2**	**T1**	10.48 a	15.75 a	0.67 a	280.0 a	280.0 a	2.59 a	22.37 a	4.26 a	1.15 a	10.34 a	0.27 a	0.07 b	0.66 a	50.28 a	18.14 a	219.28 a	1706.95 a	531.39 a	5096.05 a	34.34 a	29.17 a	23.29 a
		**T2**	9.77 ab	14.08 a	0.69 a	262.22 a	260.0 a	2.93 a	23.80 a	4.24 a	1.23 a	8.61 ab	0.30 a	0.09 ab	0.61 a	46.58 a	20.17 a	216.38 a	1641.32 a	538.91 a	4076.73 b	35.26 a	26.95 ab	18.85 b
		**T3**	9.27 ab	14.81 a	0.63 a	281.11 a	280.0 a	2.91 a	23.18 a	4.67 a	1.26 a	8.88 ab	0.32 a	0.08 ab	0.60 a	54.17 a	22.99 a	215.27 a	1809.52 a	550.81 a	4164.15 b	33.89 a	24.47 ab	19.37 b
		**T4**	8.83 b	13.53 a	0.65 a	263.7 a	255.56 a	3.15 a	23.37 a	3.92 a	1.35 a	8.26 b	0.29 a	0.10 a	0.61 a	46.52 a	26.47 a	179.42 a	1487.80 a	592.19 a	3924.94 b	31.96 a	22.38 b	22.01 ab
**Late season**	**GY9**	**T1**	6.36 a	12.85 a	0.50 a	280.0 a	200.0 ab	2.59 b	21.36 a	4.36 a	1.23 a	7.26 a	0.34 b	0.10 b	0.57 a	45.22 a	19.40 b	131.26 a	1758.32 a	518.96 a	3130.53 a	38.90 a	26.78 a	23.84 a
		**T2**	5.83 a	13.79 a	0.44 a	266.67 a	255.56 a	3.61 a	22.33 a	5.27 a	1.61 a	6.92 ab	0.38 ab	0.12 ab	0.50 ab	57.78 a	28.65 a	141.21 a	2267.78 a	695.47 a	3052.24 ab	38.97 a	24.27 ab	21.65 ab
		**T3**	5.64 a	11.64 a	0.49 a	203.33 b	190.0 b	2.95 ab	20.74 a	4.59 a	1.42 a	5.62 ab	0.39 ab	0.12 a	0.48 b	44.33 a	24.32 ab	108.11 a	2036.09 a	601.15 a	2420.21 ab	46.97 a	24.68 ab	22.46 ab
		**T4**	5.08 a	11.52 a	0.44 a	206.67 b	188.15 b	2.88 ab	19.51 a	4.9 a	1.48 a	5.14 b	0.43 a	0.13 a	0.45 b	46.56 a	29.15 a	108.59 a	2136.40 a	635.46 a	2237.06 b	46.05 a	21.84 b	20.55 b
	**ZGA**	**T1**	7.11 a	13.37 a	0.54 a	240.0 a	206.67 a	1.65 b	21.51 a	4.47 a	0.77 b	8.13 a	0.33 b	0.06 c	0.61 a	32.80 a	10.83 b	154.52 a	1880.29 a	314.01 b	3495.67 a	57.21 a	29.05 a	22.55 a
		**T2**	6.12 ab	12.99 a	0.47 a	233.33 a	215.56 a	3.51 a	21.71 a	4.44 a	1.62 a	6.92 ab	0.34 ab	0.13 a	0.53 b	39.14 a	25.86 a	143.03 a	2062.70 a	687.97 a	3044.24 a	52.62 a	26.66 ab	21.43 a
		**T3**	6.24 ab	11.95 a	0.53 a	220.0 a	208.89 a	2.01 b	23.23 a	4.83 a	0.87 b	6.26 b	0.40 a	0.07 bc	0.52 b	48.15 a	15.05 b	127.20 a	2078.60 a	359.16 b	2672.07 a	43.53 b	23.89 b	21.07 a

	**T4**	4.96 b	11.47 a	0.43 a	213.33 a	200.74 a	2.24 b	23.20 a	4.47 a	0.96 b	6.03 b	0.39 ab	0.08 b	0.53 b	44.38 a	17.91 ab	125.62 a	1889.36 a	427.64 b	2625.68 a	42.73 b	24.48 ab	20.95 a
**ZXY2**	**T1**	6.19 a	13.61 ab	0.45 a	240.0 ab	240.0 ab	2.58 a	21.88 a	4.63 a	1.18 a	7.80 a	0.34 a	0.09 a	0.57 a	46.46 a	14.40 a	171.80 a	1944.21 a	498.61 a	3402.68 a	41.71 a	34.84 a	19.81 a
	**T2**	5.57 a	14.0 a	0.40 a	280.0 a	264.45 a	3.07 a	23.86 a	4.98 a	1.28 a	7.74 a	0.36 a	0.09 a	0.55 a	45.72 a	17.21 a	171.34 a	2134.58 a	585.55 a	3376.14 a	46.69 a	34.05 a	19.65 ab
	**T3**	5.89 a	11.02 bc	0.53 a	226.67 ab	215.56 ab	2.76 a	23.90 a	3.99 a	1.16 a	5.88 b	0.36 a	0.10 a	0.53 a	38.30 a	17.87 a	161.55 a	1677.41 a	490.90 a	2617.48 ab	43.71 a	27.51 b	16.50 bc
	**T4**	5.19 a	10.30 c	0.52 a	213.33 b	207.41 b	2.57 a	22.45 a	3.67 a	1.15 a	5.48 b	0.35 a	0.11 a	0.53 a	31.29 a	18.99 a	152.22 a	1581.20 a	486.06 a	2369.27 b	50.15 a	25.78 b	15.79 c
**YXY2**	**T1**	7.11 a	16.16 a	0.45 a	340.0 a	286.67 a	3.66 a	31.10 a	5.25 a	1.61 a	9.30 a	0.32 b	0.10 a	0.58 a	42.84 a	29.54 a	216.54 a	2100.60 a	665.13 a	3999.60 a	50.48 a	22.48 a	18.50 a
	**T2**	6.47 a	14.75 ab	0.44 a	300.0 ab	273.33 a	3.34 a	27.22 ab	5.68 a	1.56 a	7.51 ab	0.39 a	0.11 a	0.51 b	61.81 a	28.47 a	188.33 ab	2305.64 a	683.71 a	3327.34 ab	37.38 b	24.07 a	17.85 a
	**T3**	5.88 a	14.29 ab	0.41 a	280.0 b	271.11 a	3.17 a	25.46 b	5.76 a	1.41 a	7.12 b	0.40 a	0.10 a	0.50 b	55.34 a	24.77 a	178.95 ab	2318.51 a	609.33 a	3089.30 b	41.95 ab	24.65 a	17.30 a
	**T4**	5.83 a	12.59 b	0.46 a	255.56 b	238.52 a	3.27 a	24.32 b	5.03 a	1.45 a	6.12 b	0.40 a	0.12 a	0.49 b	43.43 a	26.90 a	152.94 b	2022.96 a	626.40 a	2723.37 b	46.95 a	23.64 a	17.83 a
**Variety mean**	**V1**	6.88 b	13.49 ab	0.51 a	242.69 b	223.38 bc	3.19 a	19.98 b	4.81 a	1.61 a	7.07 b	0.36 a	0.12 a	0.52 b	56.81 a	26.08 a	142.38 c	1974.03 a	685.69 a	3300.36 b	36.13 a	26.92 a	23.03 a
	**V2**	7.15 b	12.77 b	0.56 a	229.44 b	214.86 c	2.53 c	21.15 b	4.28 b	1.21 c	7.28 b	0.34 b	0.09 b	0.57 a	49.66 b	23.50 a	167.82 b	1788.13 a	528.19 c	3367.57 b	38.44 a	23.37 b	20.30 b
	**V3**	7.23 b	13.56 ab	0.54 a	243.52 b	236.90 b	2.81 bc	21.11 b	4.51 ab	1.34 bc	7.70 ab	0.34 b	0.10 b	0.56 a	51.78 ab	23.12 a	194.01 a	1851.24 a	584.83 bc	3592.02 ab	37.79 a	26.60 a	18.44 c
	**V4**	7.95 a	14.49 a	0.55 a	282.82 a	268.15 a	3.13 ab	25.10 a	4.85 a	1.38 b	8.27 a	0.34 b	0.10 b	0.57 a	50.12 ab	24.68 a	195.89 a	1924.16 a	599.73 b	3800.18 a	39.03 a	24.73 b	19.38 bc

	**F value**	**Season**		410.28***	25.41***	94.49***	0.01	7.07**	1.4	40.98***	5.82*	19.8***	82.17***	103.14***	1.56	69.81***	58.76***	37.59***	120.42***	20.57***	24.19***	180.57***	353.28***	11.45**	8.47**
		**Variety**		11.96***	6.8***	2.16	19.58***	20.25***	13.66***	22.19***	4.35**	17.23***	10.21***	4.54**	23.38***	13.29***	3.34*	2.65	36.47***	2.1	13.09***	7.97***	2.45	13.61***	44.98***
		**Treatment**		23.04***	21.86***	0.03	11.16***	6.31***	4.36**	0.19	4.11**	2.98*	43.85***	14.76***	17.92***	22.23***	2.45	3.34*	10.54***	3.82*	3.41*	38.76***	3.5*	15.84***	21.2***
		**Season × Variety**		4.53**	4.85**	3.55*	1.9***	1.31	5.5**	0.96	7.05***	13.48***	1.86	3.04*	11.19***	6**	8.58***	27.71***	4.47**	4.8**	11.35***	1.68	8.23***	45.41***	15.45***
		**Season × Treatment**		0.37	3.89	2.93*	4.3**	3.51*	4.75**	1.43	2.09	4.43**	3.14*	2.61	1.36	1.96	2.61	8.6***	1.17	3.22*	5.33**	3.29*	2.79*	6.26***	2.35
		**Variety × Treatment**		0.52	1.04	0.77	1.85	0.74	1.1	0.99	0.68	0.87	1.24	0.5	2.49*	0.49	1.13	1.46	2.6*	0.46	0.73	1.41	2.91**	3.02**	5.13***
		**Season × Variety × Treatment**		0.34	1.33	0.93	0.93	1.71	2.74**	1.06	1.13	2.72**	1.47	1.63	2.71**	1.1	1.46	2.36*	1.29	1.03	2.2*	1.01	5.27***	7.15***	2.81**
**Direct-seeded rice**	**Nanning**	**GY9**	**T1**	6.69 a	22.28 a	0.30 b	390.0 a	320.0 a	3.60 a	16.39 a	9.92 a	2.21 a	10.15 a	0.45 a	0.10 a	0.46 a	79.71 a	31.41 a	151.36 a	4283.98 a	903.07 a	4455.69 a	54.27 a	29.08 a	29.59 a
			**T2**	7.05 a	14.44 b	0.49 a	271.11 b	251.11 b	2.83 ab	17.32 a	5.55 b	1.64 b	7.26 b	0.38 a	0.11 a	0.50 a	49.17 b	29.06 ab	113.34 b	2339.62 b	663.83 b	3195.42 b	51.12 a	22.96 b	28.23 a
			**T3**	6.72 a	12.36 b	0.54 a	253.33 b	246.67 b	2.75 ab	20.28 a	4.97 b	1.36 bc	6.03 b	0.40 a	0.11 a	0.49 a	48.77 b	25.02 ab	95.25 b	2060.87 b	551.75 bc	2719.93 b	42.25 a	22.09 b	28.61 a
			**T4**	6.26 a	12.91 b	0.49 a	253.70 b	230.0 b	2.22 b	19.65 a	5.40 b	1.13 c	6.39 b	0.42 a	0.09 a	0.49 a	47.46 b	19.31 b	88.27 b	2253.12 b	452.24 c	2831.89 b	51.89 a	23.41 ab	32.03 a
		**ZGA**	**T1**	7.37 a	22.17 a	0.33 c	333.33 a	300.0 a	3.02 a	17.66 b	7.38 a	1.70 a	13.09 a	0.33 b	0.08 a	0.59 a	49.79 a	24.86 a	262.12 a	2948.94 a	721.16 a	5838.53 a	61.29 a	29.04 a	22.25 a
			**T2**	7.31 a	15.78 b	0.46 b	248.89 b	244.44 b	2.17 a	21.36 ab	6.34 ab	1.01 b	8.43 b	0.4 ab	0.06 a	0.53 a	48.68 a	14.98 a	163.78 b	2561.37 ab	432.97 b	3677.51 b	52.82 a	29.18 a	22.49 a

		**T3**	7.22 a	13.36 bc	0.54 ab	265.56 b	235.56 bc	2.25 a	23.27 a	4.80 b	0.95 b	7.61 b	0.36 b	0.07 a	0.57 a	37.18 a	13.98 a	137.47 b	1885.15 b	402.31 b	3349.14 b	54.84 a	29.23 a	24.41 a
		**T4**	6.97 a	11.63 c	0.60 a	211.11 b	200.0 c	1.99 a	21.68 ab	5.48 b	0.92 b	5.23 c	0.47 a	0.08 a	0.45 b	37.68 a	13.51 a	94.61 c	2185.20 b	399.45 b	2327.92 c	60.73 a	30.85 a	24.64 a
	**ZXY2**	**T1**	7.39 a	20.14 a	0.38 b	393.33 a	360.0 a	3.45 a	18.31 a	8.14 a	1.92 a	10.09 a	0.41 a	0.09 a	0.50 a	64.68 a	36.09 a	182.44 a	3285.78 a	821.03 a	4468.29 a	50.82 a	22.82 a	24.43 a
		**T2**	6.71 ab	14.28 b	0.49 ab	264.44 b	255.56 bc	2.87 a	19.90 a	5.84 b	1.44 b	7.0 b	0.41 a	0.10 a	0.49 a	43.02 ab	26.03 a	129.96 b	2364.90 b	609.78 b	3107.02 b	62.97 a	23.54 a	24.32 a
		**T3**	6.97 a	12.43 b	0.56 a	274.44 b	272.22 b	2.60 a	19.59 a	4.70 bc	1.32 b	6.40 b	0.38 a	0.11 a	0.52 a	37.21 b	26.50 a	107.78 b	1859.34 bc	569.65 b	2861.03 b	50.28 a	21.57 a	26.57 a
		**T4**	5.76 b	11.35 b	0.51 a	252.59 b	229.63 c	2.63 a	19.76 a	4.05 c	1.34 b	5.96 b	0.36 a	0.12 a	0.53 a	29.53 b	28.71 a	102.98 b	1630.40 c	564.64 b	2631.42 b	55.27 a	19.73 a	25.91 a
	**YXY2**	**T1**	6.96 ab	14.8 a	0.47 a	340.0 a	320.0 a	3.39 a	19.65 a	5.37 a	1.72 a	7.71 a	0.36 a	0.12 a	0.52 a	50.91 a	33.03 a	128.56 a	2157.98 a	736.19 a	3460.22 a	42.98 a	22.30 a	26.90 a
		**T2**	7.52 a	12.86 a	0.59 a	304.44 ab	297.78 a	3.37 a	21.98 a	4.88 a	1.53 ab	6.44 a	0.38 a	0.12 a	0.50 a	31.19 a	27.18 a	103.35 a	1934.99 a	657.18 ab	2859.25 a	64.71 a	24.71 a	27.98 a
		**T3**	6.36 b	11.96 a	0.54 a	257.78 b	248.33 b	2.68 a	21.06 a	4.53 a	1.26 b	6.17 a	0.38 a	0.11 a	0.52 a	28.24 a	24.94 a	105.87 a	1830.8l a	542.48 b	2662.02 a	65.14 a	22.55 a	25.58 a
		**T4**	6.21 b	12.62 a	0.49 a	268.15 b	250.37 b	2.91 a	22.03 a	4.71 a	1.33 ab	6.57 a	0.38 a	0.10 a	0.52 a	31.23 a	25.07 a	122.91 a	1919.69 a	567.89 ab	2927.88 a	63.37 a	22.57 a	23.99 a
**Yulin**	**GY9**	**T1**	5.86 a	15.69 a	0.38 a	313.33 a	313.33 a	3.66 ab	16.12 b	6.51 a	2.25 a	6.93 a	0.41 a	0.14 ab	0.44 a	54.84 a	54.96 a	102.40 a	2534.18 a	871.28 a	2937.76 a	53.37 a	15.85 a	28.89 a
		**T2**	4.80 ab	13.22 a	0.36 a	302.22 ab	257.78 b	4.02 a	21.39 a	6.21 a	1.88 ab	5.13 ab	0.47 a	0.14 b	0.39 a	52.37 a	49.41 a	80.20 ab	2446.06 ab	706.63 ab	2149.81 ab	47.80 ab	14.36 a	26.60 a
		**T3**	4.14 b	9.67 b	0.43 a	257.78 b	234.44 b	2.79 b	22.0 a	4.54 b	1.24 c	3.89 b	0.47 a	0.13 b	0.40 a	72.87 a	33.10 b	61.84 b	1803.25 bc	470.85 c	1622.73 b	24.75 b	14.31 a	26.38 a

	**T4**	4.34 b	10.02 b	0.43 a	250.37 b	234.07 b	3.92 ab	22.82 a	4.27 b	1.72 b	4.03 b	0.43 a	0.17 a	0.40 a	54.05 a	45.94 a	62.75 b	1665.22 c	666.92 b	1701.93 b	36.71 ab	14.53 a	27.09 a
**ZGA**	**T1**	6.14 a	14.05 a	0.44 a	273.33 ab	260.0 a	1.94 a	14.58 b	5.79 a	1.32 a	6.94 a	0.41 a	0.09 ab	0.49 a	36.82 a	28.51 a	100.31 ab	2289.18 a	550.12 a	2961.01 a	62.41 a	19.28 a	29.65 a
	**T2**	6.07 a	12.99 ab	0.47 a	326.67 a	246.67 a	2.07 a	20.99 a	5.58 a	0.99 ab	6.42 a	0.43 a	0.08 b	0.49 a	36.56 a	20.39 a	105.66 a	2149.08 a	395.28 ab	2706.09 ab	58.80 a	19.42 a	25.58 ab
	**T3**	5.35 ab	9.99 b	0.54 a	251.11 bc	247.78 a	2.57 a	23.28 a	4.52 a	1.10 ab	4.37 b	0.45 a	0.11 a	0.44 a	27.34 a	28.30 a	70.12 b	1781.61 a	446.58 ab	1904.62 b	66.29 a	16.16 a	27.36 ab
	**T4**	4.85 b	9.93 b	0.49 a	215.56 c	201.48 b	2.02 a	23.44 a	4.64 a	0.86 b	4.43 b	0.47 a	0.09 ab	0.45 a	27.58 a	22.08 a	84.88 ab	1812.68 a	346.47 b	1927.15 b	66.53 a	16.26 a	22.71 b
**ZXY2**	**T1**	5.45 a	16.93 a	0.33 b	273.33 ab	273.33 a	3.12 a	17.20 b	7.08 a	1.81 a	8.05 a	0.41 a	0.11 a	0.48 a	57.22 a	46.96 a	112.82 a	2775.77 a	745.75 a	3441.07 a	52.79 a	15.80 a	30.52 a
	**T2**	5.09 a	12.41 b	0.41 ab	308.89 a	253.33 ab	3.3 a	25.15 a	5.34 b	1.34 b	5.73 b	0.43 a	0.11 a	0.46 a	40.52 a	36.40 ab	89.27 ab	2065.65 b	597.38 ab	2349.53 b	52.57 ab	16.23 a	26.30 a
	**T3**	5.0 a	9.50 b	0.53 a	267.78 ab	234.44 b	2.29 a	23.36 a	3.86 b	0.98 b	4.66 b	0.41 a	0.10 a	0.49 a	59.71 a	22.94 c	64.53 b	1469.41 b	384.92 c	1981.62 b	25.22 b	16.94 a	30.90 a
	**T4**	4.44 a	10.03 b	0.44 ab	240.0 b	230.37 b	2.54 a	23.20 a	4.15 b	1.09 b	4.78 b	0.42 a	0.11 a	0.48 a	55.11 a	25.27 bc	77.08 b	1550.13 b	416.29 bc	2039.44 b	28.19 ab	16.61 a	26.42 a
**YXY2**	**T1**	5.72 ab	15.71 a	0.37 b	313.33 a	313.33 a	4.0 a	20.66 b	5.79 a	1.93 a	7.98 a	0.37 a	0.12 a	0.51 a	67.80 a	37.06 a	114.42 a	2241.16 a	757.89 a	3459.17 a	33.23 a	20.55 a	30.27 a
	**T2**	6.24 a	13.66 ab	0.46 ab	315.56 a	277.78 ab	3.62 a	22.76 b	5.31 ab	1.59 ab	6.76 ab	0.39 a	0.12 a	0.50 a	65.97 a	32.26 ab	102.96 ab	2069.95 ab	634.64 ab	2924.66 ab	31.39 a	19.65 a	28.55 a
	**T3**	4.50 c	10.59 bc	0.42 ab	287.78 a	271.11 b	3.44 a	28.99 a	4.40 ab	1.19 b	5.01 bc	0.42 a	0.11 a	0.47 a	54.57 a	22.27 b	76.72 b	1836.9 ab	479.53 b	2152.44 bc	34.86 a	22.58 a	28.07 a
	**T4**	4.90 bc	9.62 c	0.51 a	261.48 a	249.63 b	3.55 a	27.84 a	3.94 b	1.29 b	4.40 c	0.41 a	0.13 a	0.46 a	50.25 a	28.51 ab	71.44 b	1494.9 b	526.36 b	1926.95 c	29.81 a	18.46 a	27.13 a

**Variety mean**	**V1**	5.73 b	13.82 a	0.43 b	286.48 a	260.93 b	3.23 ab	19.50 b	5.92 a	1.68 a	6.23 b	0.43 a	0.12 a	0.45 b	57.40 a	36.02 a	94.43 c	2423.29 a	660.82 a	2701.90 b	45.27 b	19.58 b	28.43 a
	**V2**	6.41 a	13.74 ab	0.48 a	265.69 b	241.99 c	2.25 c	20.78 b	5.57 a	1.11 c	7.06 a	0.42 a	0.08 c	0.50 a	37.70 c	20.83 c	127.37 a	2201.65 ab	461.79 c	3086.50 a	60.46 a	23.68 a	24.88 b
	**V3**	5.85 b	13.38 ab	0.46 ab	284.35 ab	263.61 b	2.85 b	20.81 b	5.40 ab	1.40 b	6.58 ab	0.40 ab	0.11 b	0.49 a	48.38 ab	31.11 b	108.36 b	2125.17 bc	588.68 b	2859.93 ab	47.26 b	19.15 b	26.92 a
	**V4**	6.05 ab	12.73 b	0.48 a	293.56 a	278.54 a	3.37 a	23.12 a	4.87 b	1.48 b	6.38 b	0.38 b	0.12 a	0.50 a	47.52 b	28.79 b	103.28 bc	1935.80 c	612.77 ab	2796.57 ab	45.68 b	21.67 a	27.31 a
**F value**	**Site**	270.72***	83.74***	20.24***	2.34	9.05**	5.68*	21.28***	17.47***	0.11	119.46***	21.37***	47.29***	55.67***	6.77*	62.7***	186.39***	29.79***	4.43*	142.03***	20.07***	189.51***	12.3***
	**Variety**	8.65***	3.11*	5.51**	5.55**	19.68***	21.23***	10.8***	8.46***	31.64***	4.23**	6.78***	48.79***	13.75***	11.43***	35.56***	18.21***	10.19***	22.82***	4.22**	8.32***	15.01***	11.5***
	**Treatment**	21.29***	121.71***	31.33***	52.87***	98.66***	6.77***	27.29***	58.47***	55.71***	86.82***	1.77	1.21	2.64	8.49***	25.31***	64.15***	56.19***	53.85***	82.34***	1.62	1.56	3.14*
	**Site × Variety**	0.94	6.59***	2.37	1.48	4.51**	4.69**	3.97*	2.32	3.9*	8.49***	0.67	11.87***	2.9*	9.65***	13.67***	10.96***	3.47*	2.75	8.54***	7.39***	14.26***	9.07***
	**Site × Treatment**	2.42	4.84**	3.08*	21.25***	6.1**	2.35	4.96**	3.65*	0.84	3.37*	1.44	1.98	1.04	4.19**	2.01	9.77***	4.38**	0.97	3.53*	1.91	0.44	5.34**
	**Variety × Treatment**	2.1*	2.44*	1.93	1.71	2.07*	0.85	1.04	3.53**	1.52	2.28*	2.62*	1.56	2.24*	0.95	1.7	3.36**	4.01***	1.39	2.1*	2.09*	1.11	1.98
	**Site × Variety × Treatment**	1.16	2.97**	2.65*	1.73	2.69*	1.42	1.09	2.3*	1.72	3.61*	1.71	3.22**	2.24*	1.41	2.4*	6.44***	2.69*	1.77	3.59**	0.69	1.38	1.03

AGB, above-ground biomass. HI, harvest index. NEP, number of effective panicles, LAI, leaf area index, SLA, specific leaf area, SB, stem biomass, LB, leaf biomass, PB, panicle biomass, SMF, stem mass fraction, LMF, leaf mass fraction, PMF, panicle mass fraction, SNA, stem nitrogen accumulation, LNA, leaf nitrogen accumulation, PNA, panicle nitrogen accumulation, SCA, stem organic carbon accumulation, LCA,

leaf organic carbon accumulation, PNA, panicle organic carbon accumulation, SCN, stem carbon-nitrogen ratio, LCN, leaf carbon-nitrogen ratio, PCN, panicle carbon-nitrogen ratio. Different lowercase letters indicate significant differences between different treatments (p<0.05). Season: early season and late season. Site: Nanning, Yulin. GY9, ZGA, ZXY5, and YXY2 respectively represent "Guiyu 9", "Zhenguiai", "Zhuangxiangyou 5" and "Yexiangyou 2". T1, 1 seedling per hill, spacing is 12.93cm. T2, 3 seedlings per hill, spacing 22.33cm. T3, 6 seedlings per hill spacing 31.67cm. T4, 9 seedlings per hill, spacing 38.80cm. Significance codes: 0 ‘***’, 0.001 ‘**’, 0.01 ‘*’, 0.05 ‘.’, 0.1 ‘ ’.

Table S5 The best models for predicting yield, above-ground biomass (AGB), and harvest index (HI) based on AICc selection.

**Model**	**Response variable**	**Models**		**Model 1**	**Model 2**	**Model 3**	**Model 4**	**Model 5**	**Model 6**	**Model 7**	**Model 8**	**Model 9**	**Model 10**	**Model 11**	**Model 12**	**Model 13**	**Model 14**	**Model 15**
**Full model 1**	**Yield**	**parameters**	**df**	15	14	16	14	16	15	13	15	14	14	13	15	14		
			**logLik**	-126.55	-127.80	-125.63	-128.13	-125.85	-127.07	-129.55	-127.21	-128.60	-128.61	-129.79	-127.51	-128.77		
			**AICc**	285.83	285.98	286.36	286.63	286.81	286.87	287.15	287.16	287.58	287.60	287.62	287.75	287.92		
			**delta**	0.00	0.15	0.53	0.80	0.98	1.04	1.32	1.32	1.75	1.77	1.78	1.92	2.08		
			**(Intercept)**	0.11	0.10	0.12	0.14	0.10	0.14	0.13	0.11	0.10	0.11	0.10	0.10	0.13		
		**growth**	**NEP**	0.13	0.10	0.11	0.12	0.14	0.12	0.14	0.08	0.12			0.14	0.14		
			**SLA**	-0.07		-0.08	-0.10	-0.09	-0.09	-0.09					-0.06	-0.09		
			**NEP:SLA**					0.05										
		**biomass**	**SB**									-0.29			-0.30			
			**LB**	0.29	0.32	0.32	0.35	0.28	0.34	0.32	0.34	0.33	0.42	0.40	0.30	0.31		
			**PB**	-1.40	-1.45	-1.38	-1.42	-1.39	-1.50	-1.49	-1.44	-1.51	-1.37	-1.37	-1.46	-1.57		
			**LMF**	-0.39	-0.41	-0.41	-0.45	-0.39	-0.43	-0.43	-0.42	-0.43	-0.47	-0.46	-0.41	-0.42		
			**PMF**															
			**PMF:SB**															

			**PB:LB**	-0.19	-0.19	-0.19	-0.20	-0.17	-0.20	-0.20	-0.19	-0.19	-0.19	-0.19	-0.19	-0.20		
			**LMF:SB**															
		**nitrogen**	**PNA**			0.37	0.45		0.50		0.30		0.41					
			**SCA**	-0.30	-0.29	-0.30	-0.30	-0.29	-0.31	-0.30	-0.29		-0.29	-0.29		-0.30		
			**PCA**	1.85	1.92	1.53	1.47	1.83	1.54	1.91	1.66	1.97	1.50	1.84	1.92	2.01		
		**carbon**	**PCA:SCA**															
		**carbon-nitr**	**SCN**	0.01	0.01	0.02		0.01	0.06		0.02	0.00	0.03	0.01	0.00	0.05		
		**ogen ratio**	**LCN**	0.04	0.04	0.06	0.06	0.05	0.06	0.04	0.05	0.02	0.04	0.02	0.03	0.04		
			**PCN**	-0.01	0.02	0.18	0.21	-0.01	0.23	-0.02	0.17	0.03	0.23	0.03	0.01	-0.02		
			**PCN:LCN**	0.19	0.19	0.22	0.24	0.19	0.24	0.21	0.21	0.18	0.21	0.18	0.18	0.20		
			**PCN:SCN**	0.09	0.11	0.08		0.09			0.10	0.11	0.09	0.11	0.10			
**Full**	**AGB**	**parameters**	**df**	13	12	13	14	14	13	14	14	13	13	12	14	13	14	15
**model**			**logLik**	361.44	360.17	361.22	362.19	361.90	360.66	361.81	361.78	360.58	360.56	359.35	361.64	360.48	361.62	362.79
**2**			**AICc**	-694.84	-694.60	-694.40	-694.02	-693.42	-693.28	-693.24	-693.18	-693.11	-693.08	-692.95	-692.91	-692.91	-692.86	-692.84
			**delta**	0.00	0.24	0.44	0.82	1.42	1.56	1.60	1.66	1.72	1.76	1.89	1.93	1.93	1.98	1.99
			**(Intercept)**	0.01	0.01	0.01	0.01	0.01	0.01	0.01	0.01	0.01	0.01	0.01	0.01	0.01	0.01	0.01
		**growth**	**LAI**									0.00			0.00			

	**NEP**	0.01	0.01	0.01	0.01	0.01	0.01	0.01	0.01	0.01	0.01		0.01	0.01	0.01	0.01
	**SLA**			0.00	0.00				0.00							0.00
**biomass**	**LB**	0.38	0.39	0.39	0.38	0.39	0.39	0.38	0.39	0.39	0.40	0.39	0.38	0.38	0.38	0.38
	**PB**	0.67	0.68	0.69	0.68	0.67	0.68	0.67	0.69	0.68	0.68	0.68	0.68	0.67	0.67	0.68
	**LMF**	-0.30	-0.31	-0.30	-0.30	-0.30	-0.30	-0.30	-0.31	-0.30	-0.31	-0.31	-0.30	-0.30	-0.30	-0.30
	**PMF**	-0.25	-0.26	-0.26	-0.25	-0.26	-0.26	-0.25	-0.26	-0.26	-0.26	-0.26	-0.25	-0.25	-0.25	-0.26
	**PMF:LB**															
	**SNA**					-0.01			-0.01		-0.01			0.01		-0.01
**nitrogen**	**LNA**	0.01			0.01	0.01		0.02				0.01	0.01	0.01	0.01	0.01
	**PNA**														0.00	
**carbon**	**SCA**	0.12	0.11	0.12	0.12	0.12	0.12	0.12	0.12	0.12	0.12	0.11	0.12	0.12	0.12	0.12
	**PCA**	0.07	0.06	0.06	0.06	0.07	0.06	0.07	0.06	0.06	0.07	0.06	0.06	0.07	0.07	0.06
	**PCA:SCA**	0.04	0.04	0.04	0.04	0.04	0.04	0.04	0.04	0.04	0.04	0.05	0.04	0.04	0.04	0.04
	**PCA:LCA**	-0.05	-0.05	-0.05	-0.05	-0.05	-0.05	-0.05	-0.05	-0.05	-0.05	-0.05	-0.05	-0.05	-0.05	-0.05
**carbon-nitr**	**SCN**	-0.01	-0.01	-0.01	-0.01	-0.02	-0.01	-0.01	-0.02	-0.01	-0.02	-0.01	-0.01		-0.01	-0.02
**ogen ratio**	**LCN**						-0.00	0.01								
	**PCN**															

			PCN:LCN								
**Full**	**HI**	**parameters**	**df**	13	14	14	14	14	14	14	13
**model**			**logLik**	-162.14	-161.66	-161.75	-161.91	-161.96	-162.01	-162.04	-163.52
**3**			**AICc**	352.33	353.70	353.88	354.19	354.29	354.39	354.45	355.09
			**delta**	0.00	1.37	1.55	1.87	1.96	2.06	2.12	2.76
			**(Intercept)**	-0.02	-0.02	-0.02	-0.02	-0.02	-0.02	-0.03	-0.03
		**growth**	**LAI**	-0.21	-0.20	-0.21	-0.21	-0.23	-0.22	-0.21	-0.20
			**tillers**					0.04			
			**NEP:SLA**						0.02		
		**biomass**	**SB**	0.09	0.12	0.05	0.09	0.06	0.10	0.07	0.08
			**LMF**	0.28	0.39	0.27	0.28	0.29	0.29	0.26	0.24
			**PMF:SB**	-0.18	-0.19	-0.19	-0.19	-0.18	-0.19	-0.19	-0.17
			**LMF:SB**							-0.03	
		**nitrogen**	**SNA**			0.13					
			**LNA**	-0.24	-0.23	-0.25	-0.25	-0.24	-0.24	-0.23	-0.22
			**PNA**	0.36	0.47	0.36	0.57	0.36	0.37	0.36	
		**carbon**	**SCA**	-0.76	-0.74	-0.81	-0.76	-0.74	-0.76	-0.76	-0.76

	**LCA**		-0.14						
	**PCA**				-0.17				0.26
	**LCA:SCA**								
	**PCA:SCA**								
**carbon-nitr**	**SCN**	-0.03	-0.02	0.09	-0.02	-0.03	-0.03	-0.03	-0.05
**ogen ratio**	**PCN**	0.18	0.22	0.17	0.28	0.17	0.18	0.18	0.00
	**PCN:LCN**	0.26	0.27	0.26	0.27	0.26	0.26	0.26	0.24
	**PCN:SCN**	0.17	0.17	0.17	0.16	0.17	0.17	0.17	0.19

Note: Full model 1 includes 20 selected predictor variables (including some interaction effects) related to growth traits, biomass accumulation, nitrogen accumulation, organic carbon accumulation, and the carbon-nitrogen ratio. The response variable is yield. Full model 2 includes 19 selected predictor variables (including some interaction effects) related to growth traits, biomass accumulation, nitrogen accumulation, organic carbon accumulation, and the carbon-nitrogen ratio, with AGB as the response variable. Full model 3 includes 16 selected predictor variables (including some interaction effects) related to growth traits, biomass accumulation, nitrogen accumulation, organic carbon accumulation, and the carbon-nitrogen ratio. The model parameters include df, logLik, AICc, and ΔAICc. The predictor variables include: number of effective panicles (NEP), tiller number, leaf area index (LAI), specific leaf area (SLA), stem biomass (SB), leaf biomass (LB), panicle biomass (PB), stem mass fraction (SMF), leaf mass fraction (LMF), panicle mass fraction (PMF), stem nitrogen accumulation (SNA), leaf nitrogen accumulation (LNA), panicle nitrogen accumulation (PNA), stem organic carbon accumulation (SCA), leaf organic carbon accumulation (LCA), panicle organic carbon accumulation (PNA), stem carbon-nitrogen ratio (SCN), leaf carbon-nitrogen ratio (LCN), and panicle carbon-nitrogen ratio (PCN).

Table S6 Names and abbreviations of above-ground traits of rice plants as used in this study

Traits	Abbreviation	Type and definition	Units
Grain yield	-	-	t ha^{-1}
Above ground biomass	AGB	Population productivity	t ha^{-1}
Harvest index	HI	Population productivity	-
Tillers	-	Population growth	10^4 ha^{-1}
Number of effective panicles	NEP	Population growth	10^4 ha^{-1}
Leaf area index	LAI	Population growth	-
Specific Leaf Area	SLA	Population growth	m^2 kg^{-1}
Stem/Leaf/Panicle biomass	SB/LB/PB	Biomass accumulation	t ha^{-1}
Stem/Leaf/Panicle mass fraction	SMF/LMF/PMF	Biomass allocation	-
Stem/Leaf/Panicle nitrogen accumulation	SNA/LNA/PNA	Nitrogen accumulation	t ha^{-1}
Stem/Leaf/Panicle organic carbon accumulation	SCA/LCA/PCA	Carbon accumulation	t ha^{-1}
Stem/Leaf/Panicle Carbon-nitrogen ratio	SCN/LCN/PCN	Carbon-nitrogen ratio	-
